# Identification and characterization of microRNAs in *Humulus lupulus* using high-throughput sequencing and their response to *Citrus bark cracking viroid* (CBCVd) infection

**DOI:** 10.1186/s12864-016-3271-4

**Published:** 2016-11-15

**Authors:** Ajay Kumar Mishra, Ganesh Selvaraj Duraisamy, Jaroslav Matoušek, Sebastjan Radisek, Branka Javornik, Jernej Jakse

**Affiliations:** 1 Biology Centre ASCR v.v.i, Department of Molecular Genetics, Institute of Plant Molecular Biology, Branišovská 31, České Budějovice, 37005 Czech Republic; 2 Slovenian Institute of Hop Research and Brewing, Plant Protection Department, Cesta Zalskega tabora 2, Žalec, SI-3310 Slovenia; 3 Department of Agronomy, Biotechnical Faculty, University of Ljubljana, Jamnikarjeva 101, Ljubljana, SI-1000 Slovenia

**Keywords:** *Humulus lupulus*, High-throughput sequencing, *Citrus bark cracking viroid*, microRNA, Viroids, Target expression

## Abstract

**Background:**

Hop (*Humulus lupulus* L.) plants are grown primarily for the brewing industry and have been used as a traditional medicinal herb for a long time. Severe hop stunt disease caused by the recently discovered *Citrus bark cracking viroid* (CBCVd) is one of the most devastating diseases among other viroid infections in hop. MicroRNAs (miRNAs) are a class of non-coding small RNAs that play important roles in gene expression regulation. To identify miRNAs in hop and their response to CBCVd-infection, two small RNA (sRNA) libraries were prepared from healthy and CBCVd-infected hop plants and were investigated by high throughput sequencing.

**Results:**

A total of 67 conserved and 49 novel miRNAs were identified. Among them, 36 conserved and 37 novel miRNAs were found to be differentially recovered in response to CBCVd-infection. A total of 311 potential targets was predicted for conserved and novel miRNAs based on a sequence homology search using hop transcriptome data. The majority of predicted targets significantly belonged to transcriptional factors that may regulate hop leaf, root and cone growth and development. In addition, the identified miRNAs might also play an important roles in other cellular and metabolic processes, such as signal transduction, stress response and other physiological processes, including prenylflavonoid biosynthesis pathways. Quantitative real time PCR analysis of selected targets revealed their negative correlation with their corresponding CBCVd-responsive miRNAs.

**Conclusions:**

Based on the results, we concluded that CBCVd-responsive miRNAs modulate several hormone pathways and transcriptional factors that play important roles in the regulation of metabolism, growth and development. These results provide a framework for further analysis of regulatory roles of sRNAs in plant defense mechanism including other hop infecting viroids in particular.

**Electronic supplementary material:**

The online version of this article (doi:10.1186/s12864-016-3271-4) contains supplementary material, which is available to authorized users.

## Background

Endogenous small RNAs (sRNAs) consist of 18–24 nucleotides (nt) which act as an important regulatory molecules to regulate the gene expression at the transcriptional and post-transcriptional levels [1]. The content of sRNA in plant cells is intriguingly variable and complex, indicating their extensive regulatory role in different biological processes. The endogenous small interfering RNAs (siRNAs) can be classified into several classes based on their biogenesis pathways, genomic loci origin and biological function, such as trans-acting siRNAs (tasiRNAs), chromatin-associated siRNAs, repeat-associated siRNAs (rasiRNAs) and natural antisense transcript-associated siRNAs (nat-siRNAs) [2]. All these siRNAs are usually processed by the action of DICER-like enzymes from double-stranded RNA (dsRNA) precursors, derived from transgenes, endogenous repeat sequences or transposons through various mechanisms [3]. In plants, mature miRNAs are short (21–24 nt) in length and most of their coding genes (*MIR* genes) consist of independent transcriptional unit with their own regulatory promoters [4]. As with animals, RNA polymerase II mediates the transcription of *MIR* genes, yielding single strand primary miRNAs (pri-miRNA), which are stabilized by the incorporation of a 5′ 7-methylguanosine cap [5] and a 3′ polyadenylate tail [6]. In plants, pri-miRNAs mainly consist of a length of 70–400 nt that can fold into self-complementary stem-loop structures. However, unlike animal miRNAs, the processing of plant miRNAs occurs in the nucleus, via two sequential step processing by a family of four Dicer-like (DCL) RNase III endonucleases [7]. The initial step includes the formation of complex with the aid of the the dsRNA-binding protein HYPONASTIC LEAVES 1 (HYL1), C2H2 zinc-finger protein SERRATE (SE), DCL and the nuclear cap-binding protein complex that cleaves the pri-miRNA near the base of the stem to yield a double-stranded intermediate miRNA: passenger strand (miRNA*) duplex known as pre-miRNA [8]. The 3′ end of the sugars of the miRNA:miRNA* duplex is methylated by HUA ENHANCER 1 (HEN1) [8] and the passenger strand is usually degraded but may also sometimes function as a miRNA [9]. The pre-miRNAs or mature miRNAs are subsequently exported to the cytoplasm by HASTY (HST) with the assistance of additional unknown factors [8]. In the cytoplasm, the mature methylated miRNA strand (guide strand) is incorporated into the effector complex known as RISC (RNA-Induced Silencing Complex) [7]. As the core component of RISC, Argonaute proteins form a complex with the miRNA and direct the effector complex for silencing the target either via RNA degradation or translational repression [10]. In plants, miRNAs are involved in the regulation of various biological processes, such as leaf, root, stem and floral organ morphogenesis and development, biosynthesis, metabolism and homeostasis, vegetative to reproductive growth phase transition, senescence, signal transduction and response to biotic/abiotic stresses [3, 9, 11], including drought [12], salinity [13], oxidative [14], hypoxia [15], nutrient stresses [16] and micronutrient deficiency or toxicity [17].

An increasing number of miRNAs have so far been discovered and deposited in the miRBase database [18] (Release Version 21.0, June 2014, http://www.mirbase.org/). Among the available 35,828 mature miRNAs, this database contains information on 19,724 plant miRNAs and miRNAs* from a total of 153 species [18]. Phylogenetic analysis of miRNAs from different plant species has suggested that many plant miRNAs are evolutionarily conserved, which is supported by observation of some common miRNAs between ferns and flowering plants [19]. Strikingly, some miRNAs are species-specific, suggesting their recent evolution as compared to conserved miRNAs [20]. These non-conserved miRNAs are more moderately expressed than conserved miRNAs and most of them have not been detected in small-scale sequencing projects [21]. High-throughput sequencing technologies are a widely used, powerful approach for the identification and the characterization of miRNAs expression profiling among different tissues according to their relative abundance at unprecedented perspectives [21]. The approach has been successfully used to discover novel miRNAs (which are generally expressed at a lower level), due to its ability to generate millions of reads randomly and independently with a determined length, which is implausible to identify by sequencing a low number of clones (sRNA library sequencing) or in situ hybridization-based methods (sRNA Northern blot and miRNA array analyses) [22]. Over the past years, high-throughput sequencing technologies have been implemented to investigate miRNAs across different plant species such as maize [23], potato [24], peanut [25], barley [26], soybean [27], Chinese cabbage [28], mulberry [29] and tobacco [30].

Hop (*Humulus lupulus* L., Cannabaceae) is a dioecious, herbaceous, climbing perennial flowering plant native to Europe, western Asia and North America. The lupulin glands of female inflorescences (also called cones or strobiles) consist of biosynthetic cells, which synthesize specific and complex metabolomes known as terpenophenolics (hop bitter acids and prenylflavonoids) and terpenoids (essential oil components), which serve as an important raw component in beer for their unique bitterness, flavour and preservative activity [31]. In addition, hop has been attributed with health benefits, including as a relaxative and sleep inducer, an anti-inflammatory agent, an estrogenic effect, antioxidant activity and cancer chemo-preventive properties [32].

Viroids are members of the smallest known pathogenic agent and cause several diseases in a wide range of host plants, including many crops [33]. They consist of single-stranded, circular, non-coding RNA with genomes ranging in size from 250 to 400 nt. Replication is solely dependent on the host transcriptional and processing machinery and cellular pathways are utilized for the transport of the resulting progeny [34]. Previous findings suggest that, similar to mature miRNAs, viroid-mediated biological actions and pathogenic activities are associated with the generation of small viroid-specific small RNAs (vsRNA), which are processed from double stranded intermediate RNA during viroid replication by the action of dicer enzymes [35]. Similar to plant endogenous small RNA, their sizes range from 21 to 24 nts in length and they have been found for *Potato spindle tuber viroid* (PSTVd) [36], *Avocado sunblotch viroid* (ASBVd) [37], *Hop stunt viroid* (HSVd) [38], *Hop latent viroid* (HLVd) and *Citrus bark cracking viroid* (CBCVd) [39]. The vsRNA regulate the host plant gene expression via target RNA degradation or translational attenuation by binding to RNA with perfect or imperfect base complementarity, thus acting as miRNA or siRNA [40]. In addition, viroid infection can cause an accumulation of host plant endogenous miRNAs, as observed in PSTVd AS1-infected tomato plants [41].

**Fig. 1:**
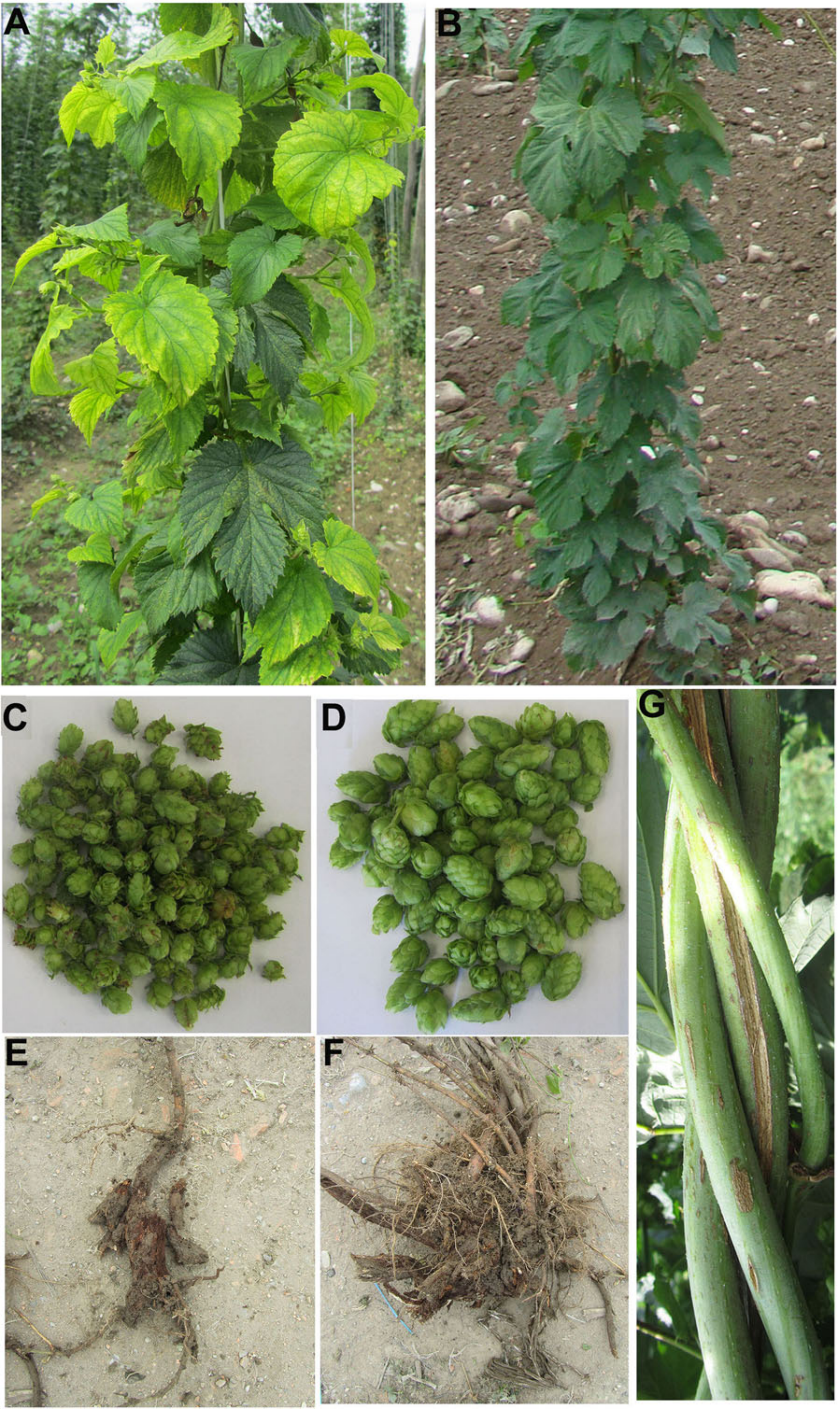
Symptoms of hop stunt disease caused by *Citrus bark cracking viroid* (CBCVd) in hop (cv. Celeia): An infected plant showing severe down curling and yellowing of leaves (**a**) compared to healthy plant leaves (**b**); infected cones reduced in size (**c**) compared to healthy cones (**d**) causing lower yield; dry root rot in infected plant (**e**) causing weaker root system, roots from healthy plants (**f**); characteristic bine cracking in infected plant (**g**)

Recently identified severe hop stunt disease caused by CBCVd (a member of the *Pospiviroidae* family) is one of the most devastating diseases, and infection caused by CBCVd disseminates rapidly by a mechanical mode of transmission, with annual incidence progression up to 20% [39]. CBCVd-infection induces dramatic morphological and anatomical changes, which include leaf epinasty, yellowing, premature flowering and a reduction in cone size, dry root rotting, stunted growth and dieback in hop (Fig. 1).

To date, no systematic studies of miRNAs in hop plant have been performed. In this study, we analysed two deep-sequenced sRNA libraries prepared from healthy and CBCVd infected hop plants, in order to characterize the miRNAs in the hop genome and investigate their expression profile in response to CBCVd-infection. In addition, the present work will lay the foundation for further systematic analysis to elucidate the other intriguing roles of miRNAs in plant-viroid interaction.

## Methods

### Plant materials and RNA preparation

Healthy and CBCVd-infected specimens of the Slovenian hop cultivar ‘Celeia’ were sampled from the experimental field of the Slovenian Institute of Hop Research and Brewing (SIRHB) and naturally infected commercial fields of local farmers, which were under the surveillance of SIRHB. The presence or absence of CBCVd-infection was confirmed by visual examination and further confirmed by recently developed CBCVd-specific RT-PCR [39]. In this study, we used our previously generated Illumina sRNA raw sequence data, which has been submitted to the NCBI Sequence Read Archive (http://www.ncbi.nlm.nih.gov/sra) under the series accession numbers SRX661829 for healthy (HP) and SRX661831, SRX661830 for CBCVd-infected (CIP) hop plants [39] for miRNA characterization and their differential expression profiling as a result of CBCVd-infection.

### Small RNA sequencing analysis

Unique tags were generated following a series of processing steps, which included removal of low quality (>30% of bases with Phred score <20) reads, trimming of reads containing adapter/primer contamination and poly-A tail using the FASTX toolkit (http://hannonlab.cshl.edu/fastx_toolkit/) [42] or CLC Genomics Server. Reads that matched rRNA, tRNA, snRNA, snoRNA, repeat sequences and other ncRNAs deposited in Rfam version 12 (http://rfam.xfam.org/) and the GenBank non-coding RNA database (http://www.ncbi.nlm.nih.gov/) were discarded after searching with the cmsearch tool. The filtered reads obtained from CIP and HP were processed using the plant-specific parameterized probabilistic-model-based miR-PREFeR core algorithm [43] by exploring the scoring system, which includes the secondary structure, the dicer cleavage site, the minimum free energy, the presence of star miRNA evidence and other criteria mentioned earlier [44]. The remaining non-redundant cleaned reads were mapped to the hop draft genome sequence [45] using bowtie-align-reads.py script, which is a wrapper for the bowtie mapper. The main script miR_PREFeR.py was used for de-novo identification of novel miRNAs, using a bowtie aligned SAM file, a hop reference genome with the following configuration parameters (GFF File = none, Precursor Len = 600 and Reads depth cutoff = 3). The accuracy and performance of miR-PREFeR, with its characteristic plant-specific scoring system, has been documented as being superior to other NGS-based miRNA prediction tools [46].

### Prediction and validation of hop miRNA target genes

The available hop transcriptome database (NCBI accession number: GAAW00000000) was downloaded and used for the prediction of potential target mRNA candidates for novel and conserved miRNAs using the psRNATarget program (http://plantgrn.noble.org/psRNATarget/) with default parameters [47]. A stringent penalty score of ≤3.0 (lower scores are better) with previously established criteria [48] was used for high specificity and low noise in miRNA target genes prediction. The predicted target transcripts of miRNAs were functionally annotated using protein databases and non-redundant nucleotide databases (http://www.ncbi.nlm.nih.gov/). Conserved domains in the target transcripts were assigned by searching them against the PFAM database using the HMMscan program. Functional categories of target gene sequences were annotated against the COG database (https://www.ncbi.nlm.nih.gov/COG/) using BLAST with a cut-off *E* value <1e^−5^. The Blast2GO program was used to perform gene ontology (GO) annotation of the target genes based on categories of biological processes, molecular functions and cellular components [49].

In order to locate the cleavage sites within the predicted target genes of miRNAs (hop-miR156, hop-miR164a and hop-miR171b), an RNA ligase-mediated rapid amplification of 5′ ends (RLM-5′ RACE) experiment was carried out using GeneRacer Kit (Invitrogen, USA) following the manufacturer’s instructions. Two micrograms of total RNA isolated from leaves was directly ligated to the 5′ RACE oligo adaptor without enzymatic pretreatment. The resultant ligated products were reverse transcribed and further PCR amplified with gene specific primers (Additional file 1: Table S1). The 5′ RACE-PCR amplicons were cloned into pGEM-T Easy Vector (Promega, USA) and sequenced for each target gene.

### Differential expression analyses of miRNAs

The frequency of miRNA read counts in HP and CIP tissues were normalized as transcripts per million (TPM) using equation (1).

1. Normalization formula: (Actual miRNA count/Total count of clean reads) × 10^6^
  Based on normalized expression analysis, the miRNA logarithmic fold-change ≥ 2.0 or fold-change ≤ −2.0, *P*-value <0.05 was used to judge the significance of the differentially recovered miRNAs between CIP and HP.
  The fold-change and *P*-value were calculated according to equations (2) and (3) respectively [50].
2. Fold change = log_2_ (normalized read counts in CIP library/normalized read counts in HP library)
3. *P* value formula:
  $$ \begin{array}{cc}\hfill \mathrm{P}\left(\mathrm{x}\left|\mathrm{y}\right.\right)={\left(\frac{{\mathrm{N}}_2}{{\mathrm{N}}_1}\right)}^{\mathrm{y}}\frac{\left(\mathrm{x}+\mathrm{y}\right)!}{\mathrm{x}!\mathrm{y}!\left(1+\frac{{\mathrm{N}}_2}{{\mathrm{N}}_1}\right){\left(1+\frac{{\mathrm{N}}_2}{{\mathrm{N}}_1}\right)}^{\left(\mathrm{x}+\mathrm{y}+1\right)}}\hfill & \hfill \begin{array}{c}\hfill \mathrm{C}\left(\mathrm{y}\le {\mathrm{y}}_{\min}\left|\mathrm{x}\right.\right)={\displaystyle \sum_{\mathrm{y}=0}^{\mathrm{y}\le {\mathrm{y}}_{\min }}\mathrm{p}\left(\mathrm{y}\left|\mathrm{x}\right.\right)}\hfill \\ {}\hfill \mathrm{D}\left(\mathrm{y}\ge {\mathrm{y}}_{\max}\left|\mathrm{x}\right.\right)={\displaystyle \sum_{\mathrm{y}\ge {\mathrm{y}}_{\max}}^{\infty}\mathrm{p}\left(\mathrm{y}\left|\mathrm{x}\right.\right)}\hfill \end{array}\hfill \end{array} $$
  N_1_ and N_2_ represent the total count of clean tags in CIP and HP libraries, respectively, x and y symbolize the normalized expression level of a particular miRNA in CIP and HP libraries, respectively, C and D are considered as the probability discrete distribution of the *P*-value inspection. The Poisson distribution model was used to examine the statistical significance of miRNA expression changes due to CBCVd-infection [50]. A positive value was considered to be up-regulation, while a negative value was considered to be down-regulation of miRNA expression levels.

### Expression analysis of miRNAs and target genes using quantitative real-time PCR (qRT-PCR)

Stem-loop qRT-PCR and end-point PCR analyses were employed to validate the predicted hop miRNAs as described previously [51]. Primer sets (miRNA-specific stem-loop RT and forward primers, and universal reverse primer) for 15 selected CBCVd-responsive novel miRNAs and eight selected CBCVd-responsive conserved miRNAs were designed (Additional file 2: Table S2) according to recommended guidelines [51]. Small RNA was isolated from leaf, root and cone tissues of HP and CIP (cv. Celeia), using a PureLink™ miRNA Isolation Kit (Invitrogen, USA). The concentration and quality of miRNAs were measured using the Qubit® miRNA Assay Kit with the Qubit® 2.0 Fluorometer and subsequently 200 ng of miRNA was reverse transcribed into cDNA using a miRNA-specific stem-loop primer and TaqMan^®^ microRNA RT kit (Applied Biosystems, USA) according to the manufacturer’s instruction. The reverse transcription (RT) reactions were carried out in a 20 μl reaction volume containing 2 μl of sRNA, 50 nM stem-loop RT primer, 0.25 mM each of dNTPs, 3.33 U reverse transcriptase, 1× reverse transcriptase buffer, 10 mM DTT and 0.25 U RNase inhibitor. The RT reactions were incubated at 16 °C for 30 min, followed by pulsed RT step of 60 cycles at 30 °C for 30 s, 42 °C for 30 s, 50 °C for 1 s. Reactions were incubated at 85 °C for 5 min to inactivate the reverse transcriptase. Uniformly expressed U6 sRNA was used as the internal control for stem-loop RT-PCR experiment. End-point PCR reaction mixtures in a final volume of 20 μl consisted of 1 μl of RT product, 0.6 units of Hot Start Ex Taq polymerase (TaKaRa Bio), 1× Taq buffer, 0.25 μM each miRNA-specific forward primer and universal reverse primer and 200 μM dNTPs mixture. The PCR amplifications were performed in a thermal cycler (Bio-Rad, USA) comprised of following steps: an initial denaturation of template at 94 °C for 2 min, followed by 40 cycles of 95 °C for 15 s and 60 °C for 1 min. The amplicon size was confirmed by 4% agarose gel electrophoresis. In addition, the end-point PCR products of five randomly selected novel miRNAs (hop-miR02, hop-miR09, hop-miR17, hop-miR28 and hop-miR42) were purified using a spin-column PCR purification kit (Promega, USA) and subsequently cloned into a TOPO cloning vector following the manufacturer’s instructions (Invitrogen, USA). Positive transformants were selected randomly (ten clones each for each novel miRNA library) and inserts were sequenced using an ABI377 sequencer (Applied Biosystems, USA) with T3 and T7 primers. The resulting sequences showed perfect sequence similarities with their corresponding mature miRNA sequences listed in the database (Table 3) and thus confirmed the specificity of the stem-loop RT-PCR amplification.

Quantitative real time PCR (qRT-PCR) was performed using a Roche LightCycler 480 instrument (Roche, USA). The PCR reaction mixture (20 μL) consisted of 2 μL of three-fold diluted RT product, 0.5 μM miRNA-specific forward primer and stem-loop reverse primer, 1× Universal SYBR^®^ Green PCR Master Mix (Invitrogen, USA). The LightCycler program consisted of an initial denaturing step at 95 °C for 10 min, followed by 45 cycles of 95 °C for 15 s and 60 °C for 60 s. Melting curves were generated using the following program: PCR products were denatured at 95 °C and cooled to 65 °C at a rate of 20 °C per second to determine the specificity of each reaction. The fluorescence signal at a wavelength of 530 nm was monitored continuously from 65 to 95 °C with an increase of temperature at a rate of 0.2 °C per second. All reactions were repeated three times and included no-template control and no reverse transcriptase as negative controls for each miRNA. The transcripts level of U6 snRNA was used as internal control to normalize quantity discrepancy of RNA input. The quantification cycle (C_q_) values were determined by the amplification curve (exponential phase) automatically by the instrument and the relative fold changes of each miRNA genes were calculated according to the minimum information for the publication of qRT-PCR experiments (MIQE) guidelines as described earlier [52].

Reverse transcriptions for 10 selected target genes were performed with an oligo (dT) primer and SuperScript® III Reverse Transcriptase (Invitrogen, USA), according to the manufacturer’s instructions, to compare their expression in leaf, root and cone tissues of HP and CIP. The resultant was diluted three-fold and 1 μL of cDNA was used as a template to perform qRT-PCR using a Roche LightCycler 480 instrument (Roche, USA) with each target gene primer (Additional file 1: Table S1). The reactions were incubated in a 96-well plate and the real-time PCR program conditions consisted of an initial denaturing step at 95 °C for 5 min, followed by 40 cycles of 95 °C for 5 s and 60 °C for 30 s. The specificity of the primers was verified by melting curves analysis following a thermal denaturing cycle of 60–95 °C at 1 °C increments with 5 s between each step. The hop specific *Hl*-GAPDH gene (GenBank Accession No. ES437736) was used as an internal control. All reactions were performed in triplicate and included no-template control and no reverse transcriptase as negative controls for each gene assay. The relative expression level of the target gene was calculated according to standard (MIQE) guidelines as described earlier [52].

## Results

### High-throughput sequencing and characterization of potential hop miRNAs

**Fig. 2:**
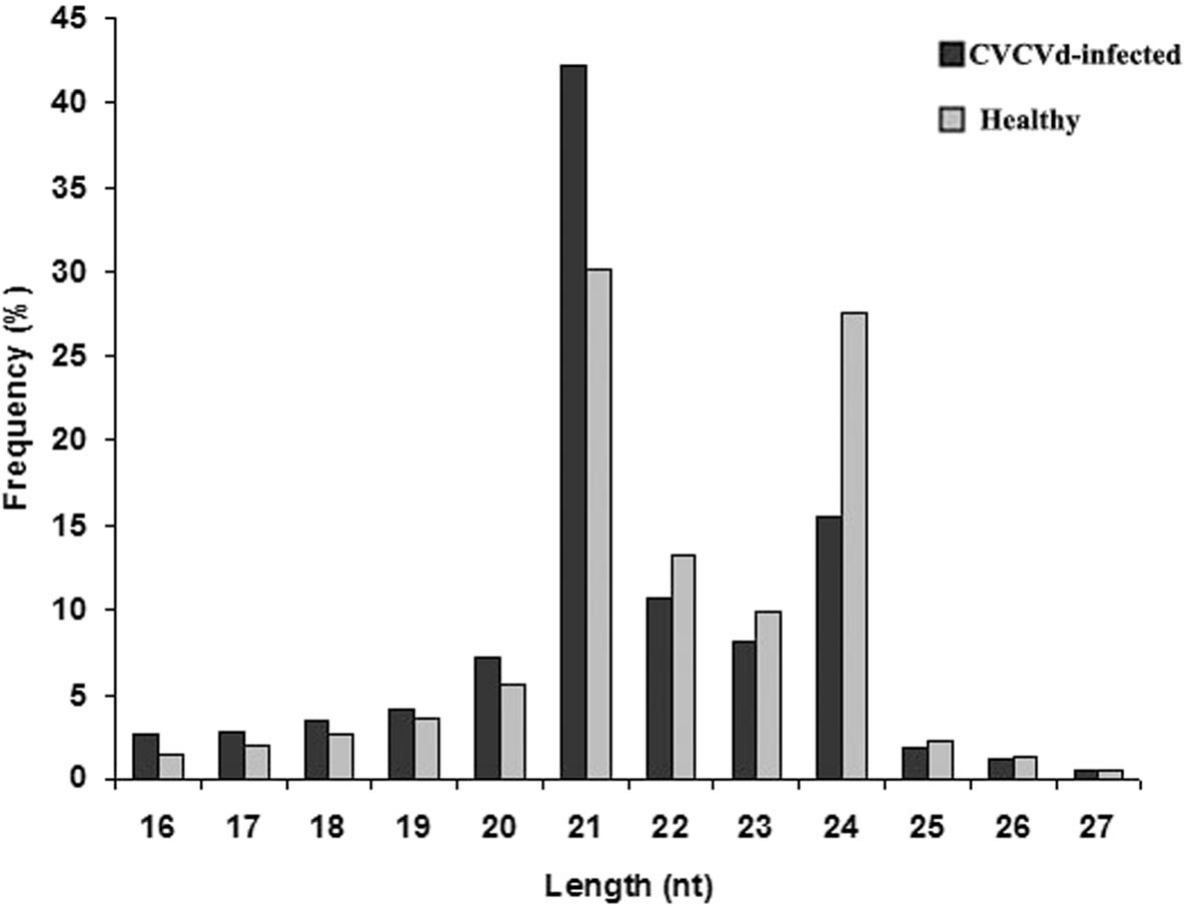
Length distribution of small RNAs in CBCVd-infected and healthy libraries in hop. The X axis represents the length of small RNAs. The Y axis represents the relative frequency

To survey sRNAs in hop and their role in plant response to CBCVd-infection, two small RNA libraries, prepared from tissues (leaf, root and cone) of HP and CIP plants, were subjected to Illumina deep sequencing [39]. The sequencing acquired 21,449,604 reads from the HP library and 68,611,723 reads from the CIP library. After removing adaptor sequences, filtering out low-quality tags and eliminating contamination produced by the adaptor-adaptor ligation, 7,253,175 and 15,907,779 clean reads were obtained from HP and CIP libraries, respectively (Table 1). The most abundant size of mapped sRNAs (>80%) was 20–24 nt in length, with 24 and 21 nt as the most frequent size groups (Fig. 2), indicating the typical size range for Dicer-derived products as reported in a previous study [7]. The genome-matched sRNA sequences were grouped into several RNA categories, such as rRNAs, tRNAs, snRNAs, snoRNAs, repeats, known miRNAs and unannotated sRNAs (Table 1). The matched known mature miRNAs comprised of 28.07% of all sequence reads in the HP library and 17.88% in the CIP library. However, the fraction of sRNA unique sequences obtained from known miRNAs accounted for only a meagre proportion of the total unique sequences in HP and CIP libraries (0.78 and 0.39%, respectively) (Table 1). The highest proportion of genome-matched sequences was unannotated sRNA sequences. These unique sequences might include some novel miRNA candidates and several siRNAs, as well as other type RNAs, which indicates that hop contains a large and diverse sRNA population.

**Table 1:** Statistics of small RNA sequences from healthy and CBCVd-infected libraries of *Humulus lupulus*

Category	Healthy library		CBCVd-infected library	
	Reads	Unique sequences	Reads	Unique sequences
Raw Reads	21,449,604		68,611,723	
Clean Reads (14–28 nt sRNA)	7,253,175 [100%]	1,192,946 [100%]	15,907,779 [100%]	4,710,976 [100%]
miRNA	2,035,988 [28.07%]	9394 [0.78%]	2,845,586 [17.88%]	18,651 [0.39%]
rRNA/tRNA/snRNA/snoRNA	123,593 [1.70%]	14,795 [1.24%]	92,650 [0.58%]	8992 [0.19%]
Un-annotation	5,093,594 [70.22%]	1,168,757 [97.97%]	12,969,543 [81.52%]	4,683,333 [99.41%]

The predicted miRNA precursor sequence length ranged from 62 to 254 nt, with an average length of 152 ± 56.14 nt (Additional file 3: Table S3) and a large percentage of them (61.20) were 100–200 nt (Additional file 4: Figure S1). The percentage composition of each nucleotide was randomly distributed in the identified hop pre-miRNAs (Additional file 3: Table S3). The U content in the identified hop pre-miRNAs, in particular, was higher than the C and G contents, which were below 20% average (Additional file 3: Table S3 and Additional file 4: Figure S1). The MFE (minimum free energy), AMFE (adjusted MFE) and MFEI (MFE index) of the identified hop pre-miRNAs (Additional file 3: Table S3 and Additional file 4: Figure S1) were consistent with previous findings [30] and indicated high-confidence prediction of miRNAs in hop.

### Identification of conserved miRNAs and their expression profile

The sRNA datasets of HP and CIP were aligned to known miRNAs sequences in the miRBase databases, resulting in the identification of 67 conserved miRNAs in hop, belonging to 40 different families (Table 2). However, the miRNAs identified in the present study were not evenly distributed among the families. The highest representation of 6 miRNAs in the MIR399 family and 4 miRNAs in families MIR169, MIR171 and MIR482 was observed. The remaining 23 miRNA families were represented by a single member. The results indicated that different members from the same miRNA families exhibited significantly different expression levels. For example, reads of miRNAs in the MIR399 family ranged from 3 to 494 and 33 to 5136 in HP and CIP groups, respectively.

**Table 2:** List of conserved miRNAs and their recoveries profiling in response to CBCVd-infection in *Humulus lupulus*

Family	miRNA name	Reference miRNA	Sequence (5′-3′)	Length	Normalized value		(log2 IS/HS)	*P*-value	Significance lable
					Healthy sample (HS)	Infected sample (IS)			
MIR156	hop-miR156	csi-miR156	UGACAGAAGAGAGUGAGCAC	20	9.926687278	46.07808544	2.214696525	1.47376E-85	**
MIR159	hop-miR159a	ath-miR159a	UUUGGAUUGAAGGGAGCUCUA	21	330.8895759	3017.517405	3.188940344	0	**
MIR159	hop-miR159c	sof-miR159c	CUUGGAUUGAAGGGAGCUCC	20	166.8234945	970.7829107	2.540826227	0	**
MIR159	hop-miR319a	ath-miR319a	UUGGACUGAAGGGAGCUCCC	20	165.5826586	242.9628926	0.553184401	3.286E-125	
MIR160	hop-miR160a-5p	ath-miR160a-5p	UGCCUGGCUCCCUGUAUGCCA	21	7.445015459	37.08877273	2.31663578	8.88414E-72	**
MIR160	hop-miR160f-5p	osa-miR160f-5p	UGCCUGGCUCCCUGAAUGCCA	21	1.102965253	8.926450386	3.016699258	4.40629E-22	**
MIR162	hop-miR162a-5p	aly-miR162a-5p	GGAGGCAGCGGUUCAUCGAUC	21	23.43801163	38.03170763	0.698352534	2.60746E-25	
MIR164	hop-miR164a	ath-miR164a	UGGAGAAGCAGGGCACGUGCA	21	0.41361197	9.115037366	4.461898728	9.32838E-28	**
MIR164	hop-miR164g-3p	zma-miR164g-3p	CACGCGCUCCCCUUCUCCACC	21	0.275741313	7.229167566	4.712442189	1.06026E-22	**
MIR166	hop-miR166a	pvu-miR166a	UCGGACCAGGCUUCAUUCCCC	21	23356.25433	12308.50642	−0.924153218	0	
MIR166	hop-miR166e-5p	aly-miR166e-5p	UGAAUGUUGUCUGGCACGAGG	21	23619.86303	12462.14195	−0.922448543	0	
MIR166	hop-miR166m-5p	zma-miR166m-5p	GGAAUGUUGGCUGGCUCGAGG	21	23621.93109	12466.91949	−0.922021883	0	
MIR167	hop-miR167-3p	ahy-miR167-3p	GAUCAUGUGGCAGUUUCAUC	20	2.48167182	159.4188604	6.005366167	0	**
MIR167	hop-miR167a-5p	ath-miR167a-5p	UGAAGCUGCCAGCAUGAUCUA	21	3.998249043	184.3752041	5.527132499	0	**
MIR167	hop-miR167c-5p	tae-miR167c-5p	UGAAGCUGCCAGCAUGAUCUGC	22	2.757413133	163.0648754	5.885986808	0	**
MIR168	hop-miR168a-5p	ath-miR168a-5p	UCGCUUGGUGCAGGUCGGGAA	21	186.8147398	189.0898786	0.017463854	4.97322E-31	
MIR169	hop-miR169b-5p	ath-miR169b-5p	CAGCCAAGGAUGACUUGCCGGC	22	3.308895759	4.90326148	0.567391856	0.00076095	
MIR169	hop-miR169c	ath-miR169c	CAAGGAUGACUUGCCGGCGAC	21	7.858627429	4.274638213	−0.878475035	0.160709021	
MIR169	hop-miR169h	ath-miR169h	UAGCCAAGGAUGACUUGCCUG	21	1.24083591	2.388768413	0.94495465	0.003574489	
MIR169	hop-miR1691-3p	gma-miR169l-3p	CGCUGGCAAGUUGUCUUUGGC	21	8.134368742	4.52608752	−0.84576591	0.191601254	
MIR171	hop-miR171	ctr-miR171	UUGAGCCGCGUCAAUAUCUCC	21	0.689353283	8.549276426	3.632486885	1.14049E-23	**
MIR171	hop-miR171b	osa-miR171b	UGAUUGAGCCGUGCCAAUAUC	21	1.102965253	28.60235863	4.696674873	1.79944E-86	**
MIR171	hop-miR171f	vvi-miR171f	UUGAGCCGCGCCAAUAUCACU	21	1.930189193	1.571558167	−0.296546594	0.681310925	
MIR171	hop-miR171f-5p	zma-miR171f-5p	CGAUGUUGGCAAGGUUCAAUC	21	0.551482627	3.143116333	2.510808328	1.38415E-07	**
MIR172	hop-miR172c	ath-miR172c	AGAAUCUUGAUGAUGCUGCAG	21	0.82722394	2.26304376	1.451914639	0.000504687	
MIR172	hop-miR172e-5p	stu-miR172e-5p	GCAUCAUCAUCAAGAUUCACA	21	3.860378386	7.857790833	1.025381501	2.04099E-08	
MIR390	hop-miR390a-3p	aly-miR390a-3p	CGCUAUCUAUCCUGAGUUUCA	21	13.64919501	94.29349	2.788342304	4.8357E-208	**
MIR390	hop-miR390a-5p	ath-miR390a-5p	AAGCUCAGGAGGGAUAGCGCC	21	12.4083591	94.23062767	2.92488371	2.9794E-215	**
MIR395	hop-miR395a	sly-miR395a	UGAAGUGUUUGGGGGAGCUCC	21	7.169274145	97.81378029	3.770138765	9.4472E-263	**
MIR395	hop-miR395d	ath-miR395d	CUGAAGUGUUUGGGGGAACUC	21	5.514826266	40.10616441	2.862436657	3.88981E-91	**
MIR395	hop-miR395e-5p	aly-miR395e-5p	AGCUCCUCUGAAGACUUCAGU	21	7.031403489	95.99077282	3.771011143	6.4322E-258	**
MIR395	hop-miR395g-5p	aly-miR395g-5p	AAUUCCUCUGAAGACUUCACU	21	18.10435	3.860378	2.229522	1.01823E-34	**
MIR396	hop-miR396b-5p	ath-miR396b-5p	UUCCACAGCUUUCUUGAACUU	21	21.64569309	80.52664046	1.89538615	1.4111E-129	
MIR398	hop-miR398b-5p	zma-miR398b-5p	GGGGUUGCCUGAGAACACAUG	21	160.619315	290.9897101	0.857322746	1.2737E-219	
MIR399	hop-miR399b	ath-miR399b	UGCCAAAGGAGAGUUGCCCU	20	0.01	115.1637824	13.49139946	0	**
MIR399	hop-miR399b-5p	aly-miR399b-5p	GGGCGUCUCUCCCUUGGCACG	21	3.584637073	127.2962115	5.150218613	0	**
MIR399	hop-miR399c-3p	ath-miR399c-3p	UGCCAAGAGGAGUUGCCCUGU	21	65.48856191	305.3851829	2.22131521	0	**
MIR399	hop-miR399d	ath-miR399d	UGCCAAAGGAGAUUUGCCCCG	21	0.01	2.07445678	7.69658979	7.5833E-08	**
MIR399	hop-miR399e	ptc-miR399e	CGCCAAAGGAGAGUUGCCCU	20	1.24083591	117.0496522	6.559664494	0	**
MIR399	hop-miR399g-5p	zma-miR399g-5p	GGGGCAUCCACUCUUUGGCAAG	22	68.10810438	322.8609097	2.245014394	0	**
MIR403	hop-miR403-3p	ath-miR403-3p	UUAGAUUCACGCACAAACUCG	21	1.378706566	140.7487494	6.673662856	0	**
MIR408	hop-miR408-3p	ath-miR408-3p	AUGCACUGCCUCUUCCCUGGC	21	6.342050206	22.6304376	1.835243278	1.574E-36	
MIR477	hop-miR473a-3p	ptc-miR473a-3p	GGAGCCUUAAGGGGAGAGUGG	21	243.4795796	330.9072876	0.442626284	1.4165E-142	
MIR477	hop-miR477a	nta-miR477a	ACUCUCCCUCAAGGGCUUCU	20	3.860378386	2.954529353	−0.385813932	0.726562864	
MIR482	hop-miR482a	ghr-miR482a	UCUUUCCUACUCCACCCAUACC	22	241.6872611	1445.142028	2.579997949	0	**
MIR482	hop-miR482b-3p	gma-miR482b-3p	UUUUCCCAACACCUCCCAUACC	22	803.2344456	2464.203205	1.617228186	0	
MIR482	hop-miR482b-5p	gma-miR482b-5p	UAUGGGGGGAUUGGGCAAAGC	21	803.2344456	2464.203205	1.617228186	0	
MIR482	hop-miR1448	ptc-miR1448	UACAUCCAACGUCUCCCACUGGG	23	0.01	0.817210247	6.352635388	0.001758949	**
MIR529	hop-miR529b	osa-miR529b	AGAAGAGAGAGAGUACAGCUU	21	2.48167182	2.325906087	−0.093519498	0.322219956	
MIR535	hop-miR535a	ppt-miR535a	UGACAACGAGAGAGAGCACGC	21	17.78531471	47.02102034	1.402619343	4.26882E-58	
MIR827	hop-miR827a	ghr-miR827a	UUAGAUGACCAUCAACAAACA	21	4.411861013	173.18571	5.294788742	0	**
MIR828	hop-miR828-3p	aly-miR828-3p	AGAUACUCAUUUGAACAAGAAA	22	4.549731669	9.177899693	1.012382578	1.61686E-09	
MIR1528	hop-miR1528	gma-miR1528	AAAAUAGAUCAAAAUAGUACUCU	23	0.82722394	2.07445678	1.326383757	0.001469896	
MIR2673	hop-miR2673a	mtr-miR2673a	CCUCUUCGUCUUCAUCUUCGG	21	0.689353283	2.388768413	1.792951557	7.85388E-05	
MIR3441	hop-miR3441-3p.1	aly-miR3441-3p.1	UGUAAUAAUGAUGUUUUGGGGUA	23	0.01	0.691485593	6.111627289	0.004806101	**
MIR4402	hop-miR4402	gma-miR4402	UUGAAUAUUAUGUGCCCCAGAC	22	0.137870657	0.817210247	2.567391856	0.011486802	**
MIR5255	hop-miR5225-3p	ppe-miR5225-3p	UCUCCCCGCGACUGAAGCCCC	21	16.68234945	20.55598082	0.301235726	3.28085E-08	
MIR6297	hop-miR6297a	ppe-miR6297a	AAUAAUUUAUGGUGUCGAAAACU	23	0.01	0.56576094	5.822120672	0.013132046	**
MIR7494	hop-miR7494	ghr-miR7494	CGUAUCAGGGAGCUUGUGGACU	22	2.343801163	7.669203853	1.710226635	1.55117E-12	
MIR7755	hop-miR7755-3p	bdi-miR7755-3p	AAUAAUUUACGAUGUCGAAAACA	23	0.551482627	0.56576094	0.03687714	0.584473414	
MIR7755	hop-miR7755-5p	bdi-miR7755-5p	AUAAUUUACAGUGUCGAAAAUA	22	0.01	0.75434792	6.237158171	0.002907523	**
MIR7815	hop-miR7815	ptc-miR7815	UCUUAAAAUAAUUGGUGGGAC	21	0.41361197	2.07445678	2.326383757	4.15138E-05	**
MIR7987	hop-miR7987	stu-miR7987	UGACAAUUUGAUCAUAUUGACA	22	0.01	1.32010886	7.044513093	3.1557E-05	**
MIR8562	hop-miR8562a	atr-miR8562a	UCGGAAUUUUCUGAAAAUUUGCA	23	1.516577223	1.257246533	−0.270551386	0.6884511	
MIR8693	hop-miR8693	gra-miR8693	AUGAAAAUCUUGAUUUUGAAGGC	23	0.137870657	2.137319107	3.954414979	6.80172E-07	**
MIR8712	hop-miR8712	gra-miR8712	ACCUAUGUGGUGAUGCAUCGCCU	23	0.41361197	0.75434792	0.866952138	0.140681352	
MIR8744	hop-miR8774	gra-miR8774	GAUUUUGAUUCAGAUAUGGAU	21	1.24083591	4.14891356	1.741421256	1.97494E-07	

The read numbers of the conserved miRNAs were used as an index for the estimation of miRNAs relative abundance levels in the HP and CIP libraries. The expression level of these conserved miRNAs were significantly different in the two libraries (Table 2) and a total of 36 conserved miRNAs were found to be CBCVd-responsive in the infected sample (*P* < 0.05, log_2_fold ≥ 2), suggesting their role in basal defence and in response to CBCVd-infection. These differentially recovered miRNAs included some highly recovered miRNAs, such as hop-miR166a and hop-miR482b-3p and also some moderately abundant miRNAs, such as hop-miR6297a (Table 2).

**Fig. 3:**
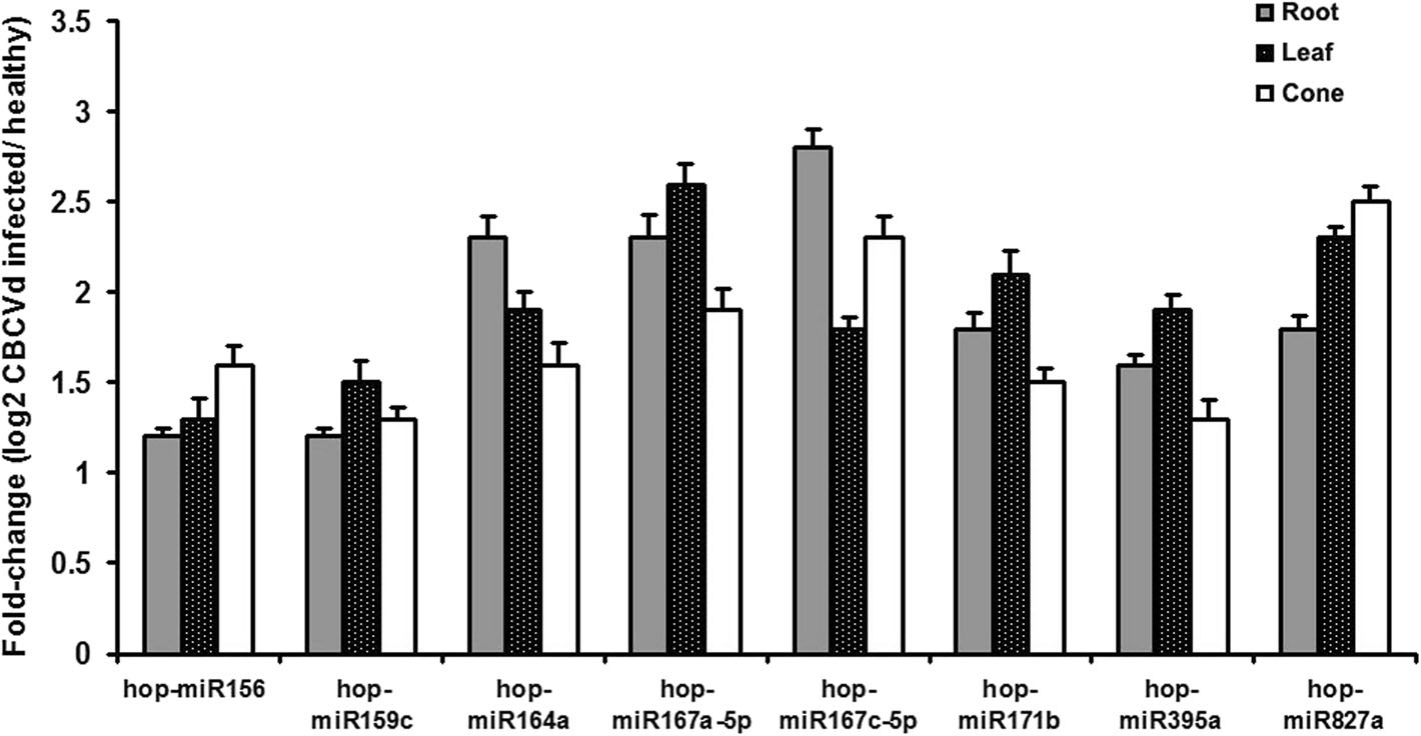
Differential recovery analysis of CBCVd-responsive 8 selected conserved miRNAs: The relative miRNA abundance in CBCVd-infected and healthy plants was evaluated using the comparative C_q_ method after normalisation to U6 snRNA as the reference. The bar graph shows log_2_ fold changes of expression level of miRNAs in CBCVd-infected samples relative to healthy samples in root, leaf and cone. miRNAs with significant change of (*P* < 0.05 and log_2_ Ratio > 1) were considered to be differentially recovered. Values are given as mean ± SD of three experiments in each group

qRT-PCR analysis of eight selected conserved miRNAs, displaying different read frequency patterns, was performed using leaf, root and cone tissues of HP and CIP. The expression levels of the eight selected conserved hop miRNAs were significantly altered in all analysed tissues as a result of CBCVd-infection (Fig. 3). Among the validated conserved miRNAs, the fold change of MIR167 was more prominent, suggesting that it is an important candidate miRNA that might play significant role in the hop stress response against CBCVd-infection. Moreover, the obtained results showed a significant correlation between qRT-PCR expression profiling and read frequencies, which suggested that the obtained sequencing profiles are both quantitatively and qualitatively reliable.

### Identification of novel miRNAs and their expression profile

Mapping of sequencing reads to the reference hop genome resulted in the identification of 49 potential miRNA candidates with a typical stem-loop structure, which were designated as hop-miR01 to hop-miR49 (Table 3). The length of the predicted novel miRNAs ranged from 19 to 23 nt, with a major peak at 21 nt, and most of them (~45%) started with a 5′ uridine, which is considered to be one of the characteristic features of miRNA. The identified hop miRNA precursor MFE values ranged from −18.30 to −139.20 kcal mol^−1^, with an average of −66.77 ± 32.58 kcal mol^−1^ (Additional file 3: Table S3), which was similar to the range reported previously for miRNA precursors of other plant species, such as maize [23] and *Arabidopsis* [53]. Sequence analysis of pre-miRNAs revealed the presence of miRNA* sequences for 49 identified novel hop miRNAs, supporting their sequence identity in our libraries and their further classification as novel miRNAs. Among the 49 novel miRNAs, only 17 had at least 0.01 TPM in both libraries, with the highest abundance, 130.31 TPM, for hop-miR41. The low abundance of novel miRNAs compared with those of conserved miRNAs in our data observed in this study was consistent with previous reports [23–30]. Normalized expression profiles of the novel miRNAs in HP and CIP libraries were compared to identify CBCVd-responsive novel miRNAs (Table 3). Among 37 differentially recovered novel miRNAs, one miRNA was found to be significantly down-regulated and 36 miRNAs were up-regulated with a more than 2.0 fold change (*P* < 0.05), in the CIP library.

**Table 3:** List of novel miRNAs and their recoveries profiling in response to CBCVd-infection in *Humulus lupulus*

miRNA name	Sequence (5′-3′)	Length	Normalized value		Fold-change (log2 IS/HS)	*P*-value	Significance lable
			Healthy sample (HS)	Infected sample (IS)			
hop-miR01	UCUGAAUAAAUUAUGGUACCGAA	23	3.033154446	3.20597866	0.079945862	0.105727	
hop-miR02	UUUCUGUUCGACUGGUAGAGA	21	4.273990356	66.57120394	3.961242702	3.32E-184	**
hop-miR03	UUGGUACCCUAAACUCAGAUU	21	18.47466799	8.046377813	−1.199137052	0.000135	
hop-miR04	UUUUUGGCACUGUAAAUUAUUCA	23	0.01	2.26304376	7.822120672	1.68E-08	**
hop-miR05	CCGAAACAUGGUCUAAACUGU	21	0.01	1.571558167	7.29605186	4.23E-06	**
hop-miR06	GUAAUUGACUGUUUGAACUCU	21	0.01	0.817210247	6.352635388	0.001759	**
hop-miR07	CUAAUGUCUCUGGCCGCGGCC	21	0.275741313	0.56576094	1.03687714	0.177719	
hop-miR08	CUAGUUUUCGGUACUGUAAACU	22	0.551482627	23.44764785	5.409983958	9.65E-75	**
hop-miR09	GGGUACCAUAUGAGCAUUAUUGA	23	1.792318536	5.154710786	1.524064425	4.58E-08	
hop-miR10	UAUAAAAUAGUUCAAAUAGACU	22	0.01	0.56576094	5.822120672	0.013132	**
hop-miR11	UCGGUGAACUCUCUUAUUCGC	21	0.01	2.26304376	7.822120672	1.68E-08	**
hop-miR12	AAUUAAAUUUGAGUUUAGGGUAC	23	0.551482627	3.708877273	2.749595188	2.75E-09	**
hop-miR13	GCUCUAAAUAACUACAAUAUACA	23	0.137870657	3.39456564	4.62183964	4.50E-11	**
hop-miR14	UUGGUACCCUAAACUCAGAUU	21	19.02615062	2.388768413	−2.993644805	7.35E-26	**
hop-miR15	UUUAGAUCUUCCUAUAUAAUAUG	23	0.01	1.005797227	6.65219567	0.000389	**
hop-miR16	AAACGUGUAGAUCUUGAAAAAA	22	0.137870657	2.137319107	3.954414979	6.80E-07	**
hop-miR17	CUCUCGAAAUACUUGUUCUACC	22	0.275741313	1.823007473	2.724933133	4.84E-05	**
hop-miR18	GAAAACAGAUUAUUUGAACUC	21	0.137870657	2.07445678	3.911346258	1.09E-06	**
hop-miR19	UAAACUAUUCGAAAUUUUCUGAA	23	0.82722394	1.382971187	0.741421256	0.052831	
hop-miR20	ACAUAUUUGGUACCAUAAACUC	22	0.137870657	2.514493067	4.188880233	3.87E-08	**
hop-miR21	UGAAUAGUUUACAGUACCGAAA	22	0.01	3.96032658	8.629475594	2.15E-14	**
hop-miR22	AGGAUAAAUCUAUGUUGCGGGUG	23	0.01	0.628623267	5.974123765	0.007944	**
hop-miR23	GUUUAAAUUUUAAGUCUUGUGGU	23	0.01	0.75434792	6.237158171	0.002908	**
hop-miR24	CACGGGUCGAGAACACUGAGAG	22	22.886529	31.1168517	0.443197422	7.12E-15	
hop-miR25	UUGGAUUCAGGGACUAUUUUU	21	0.689353283	8.737863406	3.663965116	2.80E-24	**
hop-miR26	UCCGAAUAGUUUACAGUACCGAA	23	0.01	1.194384207	6.900123184	8.62E-05	**
hop-miR27	UCGACGCUGAACCAAAUAAACAU	23	0.01	1.005797227	6.65219567	0.000389	**
hop-miR28	GAUUGGUCAAGUGGCUUACAGG	22	0.137870657	0.37717396	1.451914639	0.223324	
hop-miR29	AUCUGUAGCCAGACACAAAGG	21	0.01	0.37717396	5.237158171	0.059312	**
hop-miR30	UUUUUGGCACUGUAAAUUAUU	21	0.41361197	1.382971187	1.741421256	0.003722	
hop-miR31	GCAAAUUUUCAGAAAAUUCUG	21	0.689353283	2.64021772	1.937341466	1.63E-05	
hop-miR32	AGCCGAAAACAAAGUUCAAACUG	23	0.137870657	1.760145147	3.67430706	1.17E-05	**
hop-miR33	UGUGUAUAUUGUAGUUAUUUAGA	23	0.137870657	0.9429349	2.773842734	0.004713	**
hop-miR34	GUAAUUGACUGUUUGAACUCU	21	0.01	1.194384207	6.900123184	8.62E-05	**
hop-miR35	UUAAAAUAGAACCCUAAACCCGA	23	0.965094597	7.5434792	2.966487812	1.21E-18	**
hop-miR36	UCUGAAUUAUCAUUUAACAAA	21	0.275741313	1.69728282	2.62183964	0.000117	**
hop-miR37	CUAGUUUUCGGUACUGUAAAC	21	0.137870657	5.971921033	5.436807747	8.66E-20	**
hop-miR38	CACAUGAGCAAUAUAUUUGCA	21	2.205930506	13.89257419	2.654854698	8.36E-31	**
hop-miR39	UUUCUGUUCGACUGGUAGAGA	21	4.687602326	67.38841418	3.845578487	1.63E-183	**
hop-miR40	UCAGAAACUCCAUCGUCAACG	21	26.60903673	130.3136032	2.291999502	8.69E-246	**
hop-miR41	AUGUACAUUGUAGUUAUUUAGAG	23	0.82722394	0.628623267	−0.396082268	0.929292	
hop-miR42	UUUCUGAUCGACUGGUAGAGA	21	0.275741313	15.46413236	5.809466644	1.19E-50	**
hop-miR43	CGGGGCUUUGGGUUCAUCACC	21	4.549731669	9.115037366	1.002467109	2.19E-09	
hop-miR44	UGACAGUUUGAUCAUAUUGAC	21	0.01	5.84619638	9.191354481	6.08E-21	**
hop-miR45	AGUUUAGGGGCCUAUUUGAACU	22	0.137870657	0.691485593	2.326383757	0.027589	**
hop-miR46	UCAAACCGUCCAAACCACACCGU	23	0.01	1.50869584	7.237158171	6.99E-06	**
hop-miR47	CCCUAGACUUAGAUUUGAU	19	0.275741313	0.314311633	0.188880233	0.651715	
hopmiR48	GGGAGACUUUGUAGACAUUCAAC	23	0.01	0.251449307	4.65219567	0.162062	**
hop-miR49	CGUGGAAAACAUGUUUGAACCU	22	1.102965253	8.360689446	2.922234574	2.47E-20	**

**Fig. 4:**
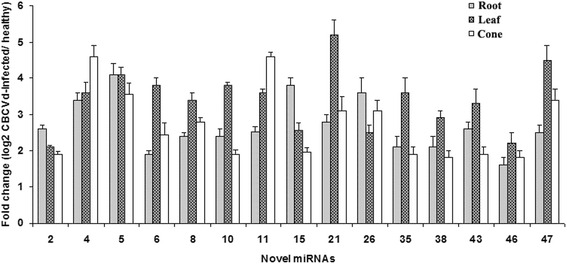
Differential expression analysis of CBCVd-responsive 15 selected novel miRNAs: The relative miRNA abundance in CBCVd-infected and healthy plants was evaluated using the comparative C_q_ method after normalisation to U6 snRNA as the reference. The bar graph shows log_2_ fold changes of expression level of miRNAs in CBCVd-infected samples relative to healthy samples in root, leaf and cone. miRNAs with significant change of (*P* < 0.05 and log_2_ Ratio > 1) were considered to be differentially recovered. Values are given as mean ± SD of three experiments in each group

The 49 putative novel miRNAs were also confirmed by stem-loop end point PCR (Additional file 5: Figure S2). In addition, stem-loop RT-PCR analysis of the expression patterns of the 15 differentially recovered novel miRNAs confirmed the changes in miRNA expression in response to CBCVd-infection in hop (Fig. 4) and were consistent with read frequencies. This analysis further supported the view that the read frequencies of the novel miRNAs were reliable both quantitatively and qualitatively. In addition, the relative expression level of nine differentially recovered novel miRNAs was significantly higher in leaves followed by roots and cones, whereas two of them were relatively higher expressed in cones, demonstrating that the relative expression level of differentially recovered novel miRNAs exhibited tissue-biased expression patterns (Fig. 4).

### Potential miRNA-target prediction and functional analysis

In order to understand the regulatory roles of the newly identified hop miRNAs, particularly the development of stunt diseases due to CBCVd-infection, target genes were scanned using the psRNATarget program [47] with default parameters against the transcript sequences of the hop genome as a reference set. About 93.89% of miRNA targets were predicted to be regulated through cleavage and 6.10% by translational repression, which is in accordance with an earlier report that mRNA cleavage is the predominant mechanism of miRNA-guided regulation [54]. Based on the extent of sequence complementarity of perfect or near-perfect pairing between miRNAs and their targets, a total of 311 unigenes were predicted as potential targets of 67 conserved miRNAs and 49 novel miRNAs, with an average of 2.68 targets per miRNA (Additional file 6: Table S4 and Additional file 7: Table S5). The MFEs for the miRNA:mRNA hybrids ranged from −2.69 to −25.53 kcal/mol. Among them, 194 unigenes were identified as potential target genes of the CBCVd-responsive miRNAs including conserved and novel miRNAs.

The potential miRNA target genes (311 different transcripts) were grouped into different gene families with a variety of biological functions, including hop growth and development, signal transduction, vegetative to reproductive phase change, homeostasis and metabolism, secondary metabolite production, protein translocation and environmental responses such as biotic/abiotic stresses. Highly-conserved miRNAs, such as miR156, miR159, miR160, miR164, miR167, miR172, miR396 and miR482, shared conserved targets of various gene families of transcription factors, such as squamosa promoter-binding-like protein (SPL), AP2-like factor, GAMYB, NAC domain containing proteins, nuclear transcription factor Y subunits (NF-Y), homeobox-leucine zipper protein, floral homeotic protein APETALA, which were phylogenetically related in hop (Additional file 8: Figure S3) with homologous miRNAs in other plants (Additional file 6: Table S4). Many of these transcription factors (such as SPL, AP2-like and GAMYB) have been shown to play an important role in vegetative to reproductive growth phase change, growth and development in *Arabidopsis* [54]. In addition to the important roles of NF-YA in floral organ identity and flowering, it might be involved in biotic and abiotic stress resistance in hop [55]. Moreover, some novel targets of both conserved and less-conserved known miRNA families were identified, which included several regulatory proteins (such as E3 ubiquitin protein, DNAJC2, CCR4). The other important predicted targets belonged to various kinds of enzymes, such as glycine dehydrogenase, cellulose synthase, glycerol-3-phosphate dehydrogenase, carboxylesterase, xylosyltransferase and β-galactosidase, which might play roles in various metabolic pathways. The identified miRNAs might also target transporters such as ABC, or transporters of sugar, heavy metals, ions and phosphate. Interestingly, non-conserved miRNAs (hop-miR482b-5p) targeted the valerophenone synthase gene involved in prenylflavonoid biosynthesis pathways in hop [56]. The predicted putative targets (141 transcripts) of novel miRNAs included a broad range of proteins with a wide array of predicted functions (Additional file 7: Table S5). For instance, hop-miR04/hop-miR13/hop-miR30 targets cytochrome P450, hop-miR20 targets RuBisCO large subunit-binding protein and hop-miR48 targets TIC110 (chloroplastic protein), which are all involved in photosynthesis [57]. The predicted targets included disease resistance proteins, such as RPM1 (hop-miR01/hop-miR39) and TMV resistance protein N (hop-miR33/hop-miR41) and several kinases, which are known to be associated with plant defense mechanisms through signaling-related processes [58, 59]. Novel targets also included several functional proteins, such as PPR-containing protein (required for normal plant development) [60], EMF1 protein (required for floral repression during vegetative development) [61], transporter proteins (such as ABC, inositol transporter 1, USO1) [62], SAND protein (endosomal maturation protein) [63], ARC5 protein (plastid division specifically in mesophyll cells) [64], GLABRA2 (trichome differentiation) [65], TT12 (controlling the vacuolar sequestration of flavonoids in the seed coat endothelium) [65]. Candidate targets of three novel miRNA families, including hop-miR05 and hop-miR47, failed to be annotated. Intriguingly, candidate targets (e.g. chalcone synthase, farnesyl pyrophosphate synthase and valerophenone synthase) of a few novel miRNAs were identified, which are widely known to be involved in prenylflavonoid and bitter acids production in hop [56].

**Fig. 5:**
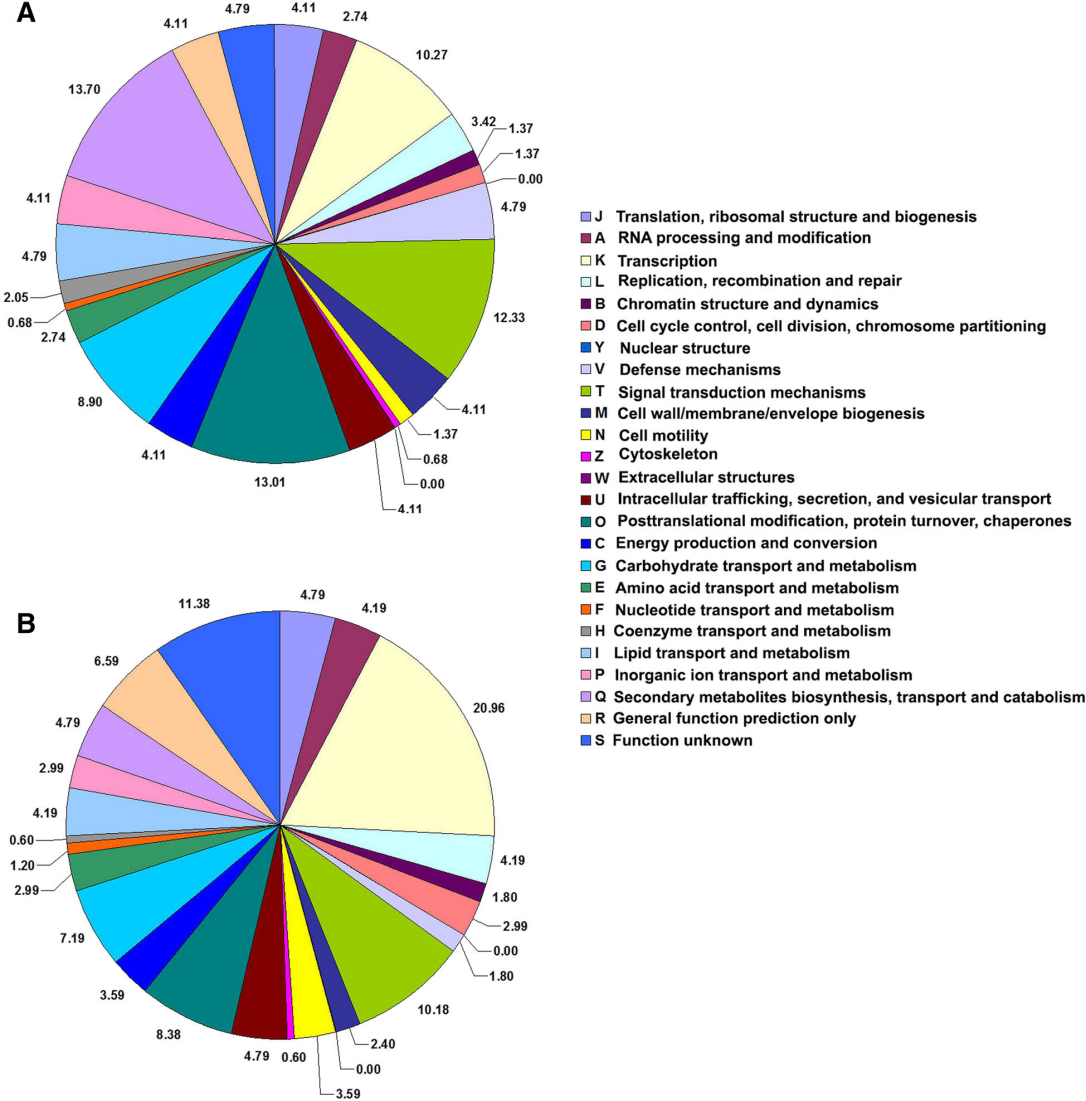
COG function classification of predicted target genes of novel miRNAs (**a**) and conserved miRNAs (**b**). Pie chart showing distribution of different functional classes represented in the predicted targets

Furthermore, COG functional classification of targets of conserved and novel miRNAs revealed that the highest proportion of genes was involved in the transcription process, while other target transcripts encode a wide range of proteins, such as those involved in RNA processing and modification, post-translational modification and protein turnover, signal transduction mechanisms, carbohydrate, inorganic ion and amino acid transport and secondary metabolite biosynthesis (Fig. 5). In addition, we found that 4.8 and 11.4% of the predicted targets of novel miRNAs and conserved miRNAs, respectively, were scantily characterized genes, suggesting that these miRNAs might have naive roles in hop.

**Fig. 6:**
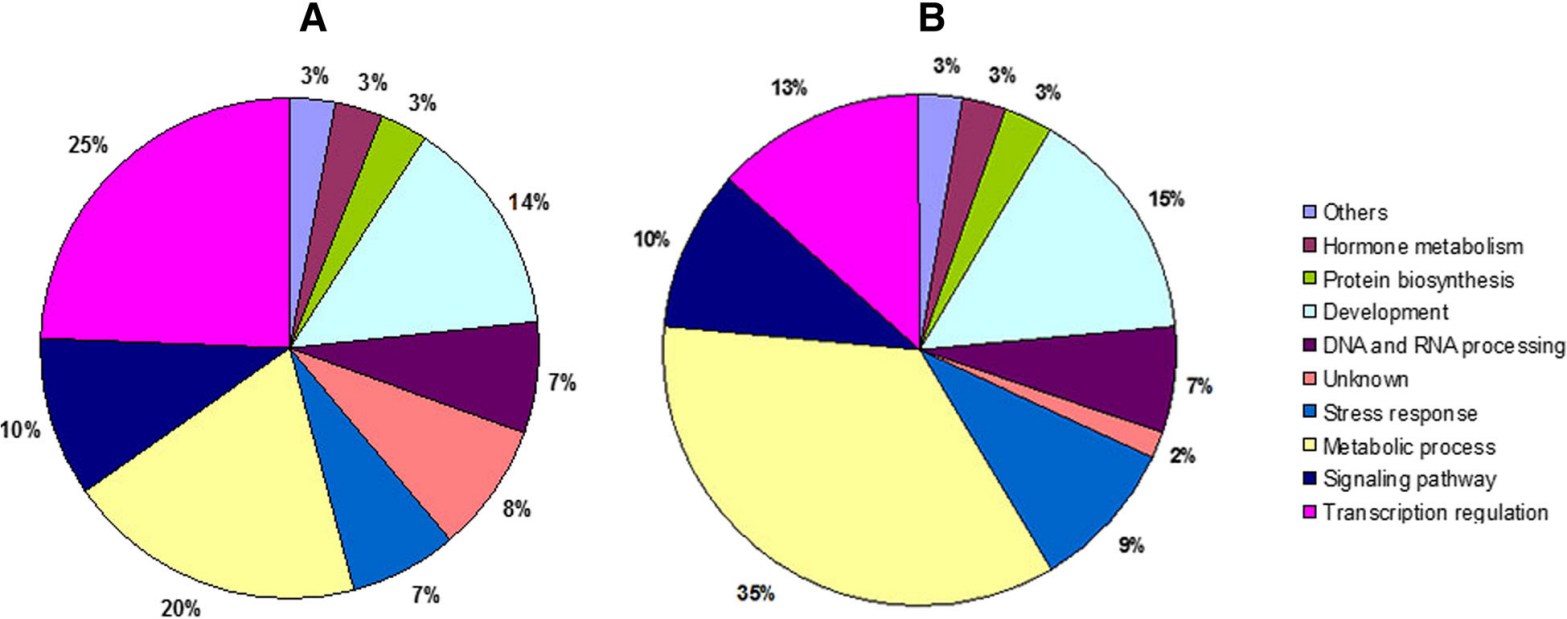
Percentage distribution of the predicted target genes of CBCVd-responsive conserved miRNAs (**a**) and novel miRNAs (**b**) in various categories

Comprehensive annotation of transcripts based on ontological definitions of the GO terms categorized the predicted targets of the miRNAs into a wide range of regulatory functions, and some specific biological processes, such as metabolism, biosynthesis and gene expression/transcription (Additional file 9: Figure S4). In order to gain an insight into the role of miRNAs in the pathogenesis of CBCVd-infection, the target genes of CBCVd-responsive miRNAs were grouped into 10 categories (Fig. 6) based on GO functional annotations using their ontologies in *Arabidopsis*. The first category of target genes of CBCVd-responsive miRNAs was represented by an array of transcriptional factors possibly involved in the regulation of gene expression. In the second category, target genes were associated with the metabolic process, suggesting that CBCVd-infection could alter the various metabolic processes in an infected plant. Target genes involved in signaling pathways belonged to the third category, indicating the role of signaling pathways in the CBCVd-infection process. The other categories included DNA and RNA processing, hormone metabolism, stress response, development and others biological processes (Fig. 6).

### Expression profile and experimental validation of target transcripts

**Fig. 7:**
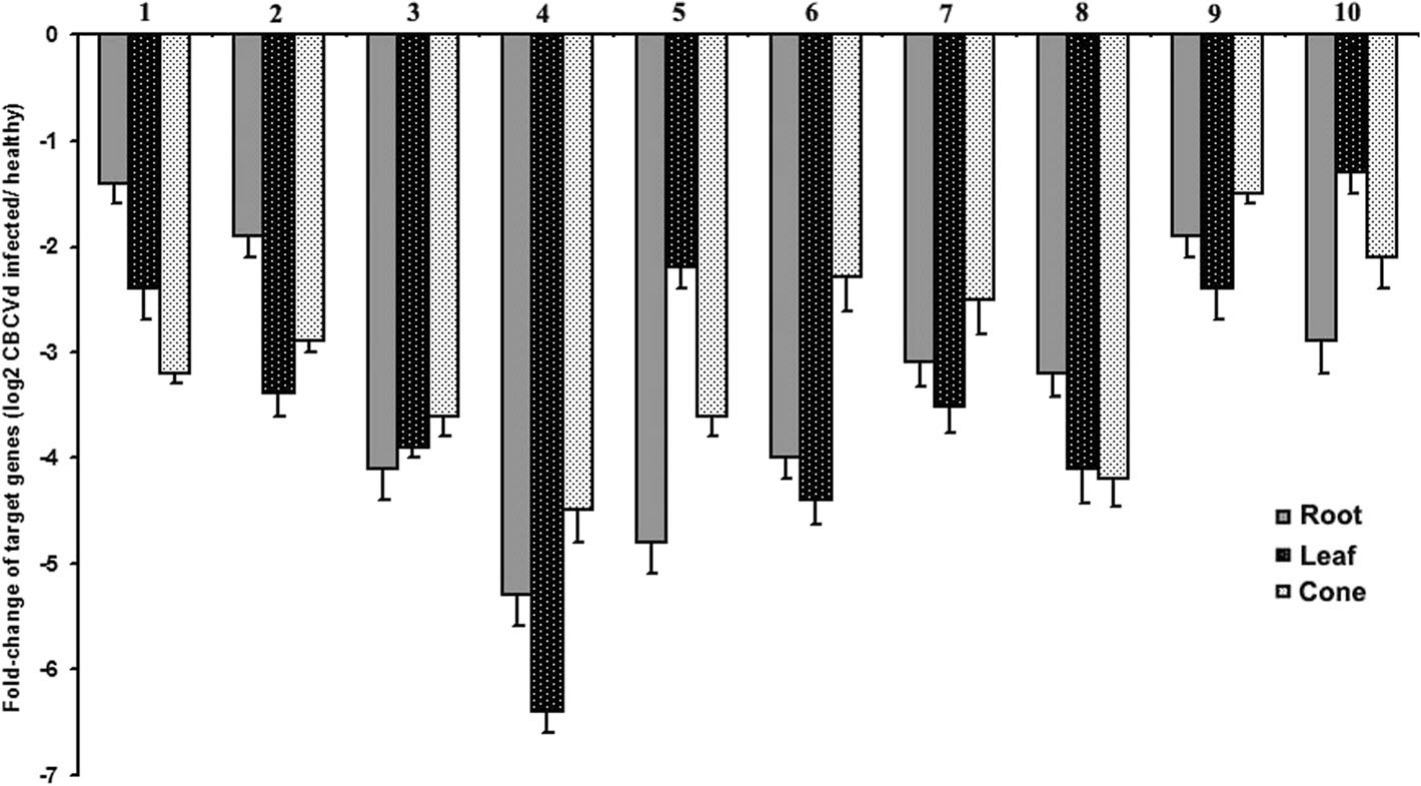
Differential expression profile of selected miRNA target genes: The relative gene expression was evaluated using the comparative C_q_ method taking *Hl*-GAPDH (GenBank Accession No. ES437736) as the reference gene. The log_2_ values represent the ratio of the expression level of target genes in CBCVd-infected samples relative to healthy samples in root, leaf and cone. The predicted target genes used in this analysis were (1) Squamosa promoter-binding protein 15 (hop-miR156 Target); (2) GAMYB Transcriptional factor (hop-miR159c Target); (3) NAC domain protein NAC1 (hop-miR164a Target); (4) Cysteine protease (hop-miR167a-5p Target); (5) mediator of RNA pol II subunit 26b (hop-miR167c-5p Target); (6) GRAS family TF (hop-miR171b Target); (7) pre-mRNA-splicing factor RNA helicase PRP1 (hop-miR395a Target); (8) Glycerol-3-phosphate dehydrogenase (hop-miR827a Target); (9) disease resistance protein RPM1-like (hop-miR02 Target); (10) cellulose synthase A catalytic subunit 4 (hop-miR41 Target). Target genes with a significant change of (*P* < 0.05 and log_2_ Ratio > 1) were considered to be differentially expressed. Values are given as mean ± SD of three experiments in each group

The expression profiles of 10 selected target transcripts of CBCVd-responsive differentially recovered miRNAs were investigated via qPCR. The result indicated that the expression of target genes had a negative correlation with expression of their corresponding miRNAs and thus confirmed their interaction in response to CBCVd- infection. However, the fold change values of CBCVd-responsive targeted genes were not uniform across the analysed infected tissues in hop (Fig. 7).

**Fig. 8:**
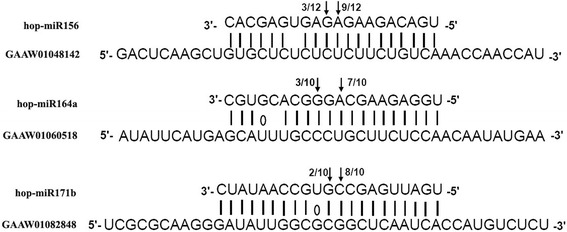
Mapping of target mRNA cleavage sites of hop-miR164a, hop-miR171b and hop-miR41 by RNA ligase-mediated 5′RACE. The positions of cleavage sites are indicated by *arrows*, and numbers above the arrow represent the proportion of sequenced clones. Watson-Crick pairing (*vertical dashes*) and G:U wobble pairing (*circles*) are indicated

The cleavage sites within the target transcripts of hop-miR156, hop-miR164a and hop-miR171b were validated by 5′ RLM-RACE analysis. Sequence analysis of the cleavage products by 5′ RACE showed hop-miR164a-mediated cleavage in the NAC domain protein transcript (GAAW01060518) at sites opposite the 10th and 12th nucleotides from the 5′ end of miRNA, and hop-miR156 and hop-miR171b mediated cleavage in Squamosa promoter-binding-like protein 15 (GAAW01048142) and GRAS family transcription factor (GAAW01082848), at sites opposite the 10th and 11th nucleotides from the 5′ end of miRNA, respectively (Fig. 8).

## Discussion

Hop is an economically important crop for the brewing industry and over the last few years has gained increasing attention due to its potential applications to pharmaceutical industries [31, 32]. In spite of that, hop miRNAs and their association with various biological processes are widely unexploited, although in the past few years several conserved miRNAs and species-specific miRNAs have been identified in various plant species with the aid of high-throughput sequencing methods. In the present study, a high-throughput deep sequencing method was used to identify conserved and novel miRNAs in hop. Their response to CBCVd-infection was also investigated, delivering valuable information for gaining in-depth knowledge of the function and regulatory mechanisms of miRNAs in the CBCVd-defence response. Comparison of sequencing data of the two libraries showed that sRNAs of 21-nt and 24-nt formed two major classes, consistent with the distribution patterns of sRNAs in other plant species [29, 30]. However, their distributions were significantly different between two analysed libraries. The CPI library had a higher abundance of the 21-nt class of sRNAs, while the 24-nt class of sRNAs was skewed in the HP library. This may indicate either an induction of the 21 nt class or a repression of the 24 nt class of sRNAs in response to CBCVd infection. This observation is in accordance with some previous reports showing the over accumulation of the 21-nt class of sRNAs in response to viroid and virus infection in plants [66, 67]. The different distribution patterns of 21-nt and 24-nt classes of sRNAs provided explicit evidence to support the possibility of the involvement of multiple RNA-silencing pathways and their cross interaction [68] due to CBCVd-infection. In plants, endogenous 24-nt small interfering RNAs (siRNAs) are mostly derived from transposons, intergenic and repetitive genomic regions, which, after incorporation with Argonaute 4 (AGO4), triggers DNA methylation [69]. These modifications reinforce transcriptional repression or silencing of a subset of transposons and genes that harbour or reside adjacent to transposons or repeats in *Arabidopsis* [69]. The reduced 24-nt sRNA levels in the CIP library suggests a lower level of DNA methylation at specific loci in response to CBCVd-infection, which could enhance the resistance or susceptibility to CBCVd or other viroid infection. However, further investigation is needed in this area to prove these assumptions.

Analysis of sRNA reads from these two hop libraries revealed the existence of 67 conserved miRNAs belonging to 40 miRNA families, and 49 novel miRNAs, further supported by the presence of their corresponding star sequences in both libraries. Furthermore, the characteristic features of pre-miRNAs (such as length distribution, MFE) and miRNAs (such as size distribution, nucleotide composition of miRNAs and prevalence of 5′-uridine) were similar to those observed in other plants [28–30]. MFEI is considered as an another valuable criterion to distinguish miRNAs from other types of coding and non-coding RNAs. The pre-miRNAs predicted had high MFEI values (1.20 ± 0.32), higher than other types of noncoding RNAs, e.g. tRNAs (0.64), rRNAs (0.59) and mRNAs (0.62–0.66) [70]. Taken together, these data indicated high-confidence prediction of miRNAs in hop.

Several previous studies have shown that group of well-conserved miRNAs (e.g. MIR156, MIR159, MIR164, MIR166, MIR167, MIR169, MIR171, MIR172, MIR319 and MIR396) often retain homologous target interactions and perform similar molecular functions across plant phyla in the course of evolution and adaptation [71]. These evolutionarily conserved miRNAs regulate targets such SPL, MYB, NAC, HD-ZIPIII, ARF, NF-Y, SCL, AP2 and TCP. These transcriptional factors are involved in metabolic processes, growth and development and might involve in the cellular adaptive responses to adverse circumstances [72]. The conserved nature of these miRNAs and their functionally conserved target transcriptional factors highlights their phylogenetic distribution and versatile functions among diverse plant lineages. The target genes of other well- and less-conserved miRNAs were predicted to be implicated in various functions, such as protein degradation, defence mechanism, basic metabolic processes, regulation of ion homeostasis, lipid and sugar translocation, signal perception and transduction, and transposable elements, suggesting the important roles of miRNAs in the regulation of a wide range of biological activities in hop. The expression levels of novel hop miRNAs were very low, which is in accordance with previous reports that lineage-specific miRNAs are usually tend to be expressed at a low level [25]. Intriguingly, some novel targets were identified that play specific roles in trichome differentiation and bitter acids and prenylflavonoids biosynthesis in hop. Further studies implementing on miRNA directed gene regulation of the prenylflavonoids biosynthesis pathway are necessary to establish their relationship and functional importance in hop.

Accumulating data suggest that plant disease resistance gene families are comprised of thousand of members, which are usually considered to be putative target genes of sRNAs [73]. The mechanism of CBCVd-associated disease could therefore be better understood by analysing sRNAs and the expression profiles of target genes in hop with and without CBCVd-infection. Comparison of the two sequence libraries data showed that almost half of the conserved miRNA families (MIR156, MIR159, MIR160, MIR164, MIR167, MIR168, MIR171, MIR172, MIR390, MIR395, MIR399, MIR403, MIR482, MIR827, MIR3441, MIR5255, MIR6297, MIR7755, MIR7815, MIR7987 and MIR8963) and 37 novel miRNAs exhibited altered expression due to CBCVd-infection. In addition, miRNAs of the same family (MIR171, MIR482) shared a high degree of sequence similarity and were differentially regulated, suggesting that gene expression programming was controlled by different regulators [74]. In the present study, hop-miR156 was significantly induced in CIP and squamosa-promoter binding-like protein (SPL) was predicted as its target gene. SPL transcription factor family proteins are known to play an important role in flower and fruit development, plant architecture and phase transition from juvenile to adult and to flowering [75]. The previous study showed that the interaction of mir156-SPL could directly regulate flowering through activating MADS box genes, resulting in the loss of apical dominance, smaller leaves development and the formation of more leaves with shorter plastochron lengths [75]. Hop-miR172e-5p was predicted to target AP2 domain-containing transcription factor, which has a pivotal role in the regulation of floral organ identity and flowering time [76]. The differential expression of miR156 and miR172 might therefore affect the growth and development process and could be responsible for hop stunt disease. In plants, a group of miRNAs modulates the fine-tuned genes associated with multiple hormonal signaling pathways [77]. Our results showed that some miRNAs that target genes involved in hormone signaling and metabolism were differentially recovered between healthy and infected hop plants. For instance, miR160/miR167, identified as dynamic components of the auxin response pathway [77] were up-regulated in the CBCVd-infected plants. Moreover, hop-miR159c, hop-miR164a and hop-miR171, associated with gibberellic acid, abscisic acid and salicylic acid pathways, respectively, by regulating different transcription factors (GAMYB, NAC and GRAS families) were found differentially recovered in healthy and CBCVd-infected hop (Fig. 7). It has been reported that the NAC protein plays a role in developmental processes such as the formation of the shoot apical meristem, floral organs and lateral shoots, the GAMYB protein in anther development, stem elongation, floral initiation and seed development and the GRAS protein in seed germination, stem and root elongation and fertility [78]. In addition, hop-miR396b-5p was predicted to target the gene coding the pentatricopeptide repeat-containing superfamily protein, which is known to be involved in the abiotic stress response by integrating salicylic acid-dependent, abscisic acid-dependent or independent signaling pathways (Additional file 6: Table S4) [79]. It is therefore more likely that CBCVd-infection could modify the hormone signaling pathway, leading to reprogramming and alteration of hop growth and development and inducing symptoms. In this study, several CBCVd-responsive miRNAs involved in defense mechanisms were identified. For example, hop-miR159c and hop-miR828-3p were predicted to target CCR4-associated factor 1 and serine/threonine-protein kinase abkC, respectively, which have been reported to be involved in signaling cascades during host pathogen interaction, the subsequent activation of plant defense mechanisms and developmental control [80]. Moreover, the present work also showed that some differentially recovered miRNAs target the genes, which regulate sugar, protein and lipid metabolism and their transportation.

In contrast to miRNAs, the length of siRNAs ranges from 21 to 25 nt and they are processed from dsRNA precursors by RNA-dependent RNA polymerases and Dicer-like (DCL) proteins and RNA polymerase IV [2]. It has been reported that 22 nt forms of miRNAs (miR173, miR319, miR828 and miR771), along with tasiR2140, are important for triggering secondary siRNA production and the target genes of these siRNAs have a wide range of functions in hormone networks, metabolism, growth and development in plants [73]. It would thus be intriguing to explore the role of hop-miR828-3p in triggering production of siRNAs, and their pathways and functions in the context to CBCVd-infection in hop.

## Conclusions

In conclusion, global transcriptional profiles of sRNAs were investigated in hop with and without CBCVd-infection. The identified miRNAs from hop could assist in identifying the miRNA-based regulatory system, together with their role in the biosynthesis pathway of prenylflavonoids and bitter acids production in hop. The differential expression profiles of miRNAs and their involvement in complex regulatory networks reveal their putative roles underlying CBCVd pathogenicity and the symptoms they cause in hop plants. Since the hop genome is not completed, we speculate that our analysis did not predict all hop miRNAs.

## Additional files

Additional file 1: Table S1. Details of primers designed for RT-PCR amplification and 5′-RACE amplification of selected target genes. (DOC 49 kb)
Additional file 2: Table S2. Details of primers designed for RT-PCR amplification of 50 novel and 8 conserved miRNAs identified in this study. (DOC 143 kb)
Additional file 3: Table S3. Information of predicted precursor sequences and characteristics of novel and conserved miRNAs in hop. (XLS 75 kb)
Additional file 4: Figure S1. Characterization of miRNAs identified in hop. (A) length distribution of pre-miRNAs; (B) distribution of nucleotides; (C) MFE and AMFE; and MFEI (D). (JPG 64 kb)
Additional file 5: Figure S2. End-point PCR validation by agarose gel electrophoresis for amplicons of the 50 novel miRNAs identified in this study. Small RNA was isolated from CBCVd-infected leaf and amplified with miRNA specific forward primer and a universal reverse primer. (JPG 60 kb)
Additional file 6: Table S4. Putative predicted targets for the 66 conserved miRNAs in hop. (XLS 55 kb)
Additional file 7: Table S5. Putative predicted targets for the 50 novel miRNAs in hop. (XLS 41 kb)
Additional file 8: Figure S3. Phylogenetic tree of APETALA 2 (A), NF-Y (B), NAC (C), SPL (D) transcriptional factor in hop. Multiple sequence alignment of the full length of protein sequences was performed using the ClustalW program. Phylogenetic trees were constructed via the maximum composite likelihood substitution model using MEGA (version 6.0). Bootstrapping was performed 1000 times to obtain support values for each branch. The scale bar represents the amino acid substitution rate. The tree has been rooted using the single representative of monocotyledonous plant. (JPG 324 kb)
Additional file 9: Figure S4. Distribution of GO categories for the predicted target genes of conserved and novel miRNAs identified in hop. The vertical axis represents the GO category, including biological processes, cellular components and molecular functions and the horizontal axis represents the number of genes. (JPG 158 kb)

## Abbreviations

AMFE: Adjusted MFE
CBCVd: *Citrus bark cracking viroids*
CIP: CBCVd-infected plants
HP: Hop healthy plant
MFE: Minimum free energy
MFEI: MFE index
microRNA: miRNA
qRT-PCR: quantitative real time PCR
sRNA: small RNA
TPM: Transcript per million

## References

[1] Chen X (2009). Small RNAs and their roles in plant development. Annu Rev Cell Dev Biol.

[2] Lu C, Tej SS, Luo S, Haudenschild CD, Meyers BC, Green PG (2005). Elucidation of the small RNA component of the transcriptome. Science.

[3] Vaucheret H (2006). Post-transcriptional small RNA pathways in plants: mechanisms and regulations. Genes Dev.

[4] Reinhart BJ, Weinstein EG, Rhoades MW, Bartel B, Bartel DP (2002). MicroRNAs in plants. Genes Dev.

[5] Xie Z, Allen E, Fahlgren N, Calamar A, Givan SA, Carrington JC (2005). Expression of Arabidopsis MIRNA genes. Plant Physiol.

[6] Zhang BH, Pan XP, Wang QL, Cobb GP, Anderson TA (2005). Identification and characterization of new plant microRNAs using EST analysis. Cell Res.

[7] Margis R, Fusaro AF, Smith NA, Curtin SJ, Watson JM, Finnegan EJ, Waterhouse PM (2006). The evolution and diversification of dicers in plants. FEBS Lett.

[8] Xie M, Zhang S, Yu B (2015). microRNA biogenesis, degradation and activity in plants. Cell Mol Life Sci.

[9] Voinnet O (2009). Origin, biogenesis, and activity of plant microRNAs. Cell.

[10] Baumberger N, Baulcombe DC (2005). Arabidopsis ARGONAUTE1 is an RNA Slicer that selectively recruits microRNAs and short interfering RNAs. Proc Natl Acad Sci U S A.

[11] Duraisamy GS, Mishra AK, Jakse J, Matousek M (2015). Computational Prediction, Target Identification and Experimental Validation of miRNAs from Expressed Sequence Tags in *Cannabis sativa.* L. Res Rev: J Bot Sci.

[12] Wang TZ, Chen L, Zhao MG, Tian QY, Zhang WH (2011). Identification of drought-responsive microRNAs and their targets in *Medicago truncatula* by genome-wide high-throughput sequencing and degradome analysis. BMC Genomics.

[13] Ding D, Zhang L, Wang H, Liu Z, Zhang Z, Zheng Y (2009). Differential expression of miRNAs in response to salt stress in maize roots. Ann Bot.

[14] Shukla LI, Chinnusamy V, Sunkar R (2008). The role of microRNAs and other endogenous small RNAs in plant stress responses. Biochim Biophys Acta.

[15] Moldovan D, Spriggs A, Yang J, Pogson BJ, Dennis ES, Wilson IW (2010). Hypoxia-responsive microRNAs and trans-acting small interfering RNAs in Arabidopsis. J Exp Bot.

[16] Zhao M, Ding H, Zhu JK, Zhang F, Li WX (2011). Involvement of miR169 in the nitrogen-starvation responses in Arabidopsis. New Phytol.

[17] Valdes-Lopez O, Yang SS, Aparicio-Fabre R, Graham PH, Reyes JL, Vance CP, Hernández G (2010). MicroRNA expression profile in common bean (*Phaseolus vulgaris*) under nutrient deficiency stresses and manganese toxicity. New Phytol.

[18] Kozomara A, Griffiths-Jones S (2014). miRBase: annotating high confidence microRNAs using deep sequencing data. Nucleic Acids Res.

[19] Axtell MJ, Bartel DP (2005). Antiquity of microRNAs and their targets in and plants. Plant Cell.

[20] Moxon S, Jing R, Szittya G, Schwach F, Rusholme Pilcher RL, Moulton V, Dalmay T (2008). Deep sequencing of tomato short RNAs identifies microRNAs targeting genes involved in fruit ripening. Genome Res.

[21] Motameny S, Wolters S, Nürnberg P, Schumacher B (2010). Next Generation Sequencing of miRNAs-Strategies, Resources and Methods. Genes (Basel).

[22] Jagadeeswaran G, Zheng Y, Sumathipala N, Jiang H, Arrese EL, Soulages JL, Zhang W, Sunkar R (2010). Deep sequencing of small RNA libraries reveals dynamic regulation of conserved and novel microRNAs and microRNA-stars during silkworm development. BMC Genomics.

[23] Zhang L, Chia JM, Kumari S, Stein JC, Liu Z, Narechania A, Maher CA, Guill K, McMullen MD, Ware D (2009). A Genome-Wide Characterization of MicroRNA Genes in Maize. PLoS Genet.

[24] Lakhotia N, Joshi G, Bhardwaj AR, Katiyar-Agarwal S, Agarwal M, Jagannath A, Goel S, Kumar A (2014). Identification and characterization of miRNAome in root, stem, leaf and tuber developmental stages of potato (*Solanum tuberosum* L.) by high-throughput sequencing. BMC Plant Biol.

[25] Chi X, Yang Q, Chen X, Wang J, Pan L, Chen M, Yang Z, He Y, Liang X, Yu S (2011). Identification and Characterization of microRNAs from Peanut (*Arachis hypogaea* L.) by High-Throughput Sequencing. PLoS ONE.

[26] Lv S, Nie X, Wang L, Du X, Biradar SS, Jia X, Weining S (2012). Identification and characterization of microRNAs from Barley (*Hordeum vulgare* L.) by high-throughput sequencing. Int J Mol Sci.

[27] Shamimuzzaman M, Vodkin L (2012). Identification of soybean seed developmental stage-specific and tissue-specific miRNA targets by degradome sequencing. BMC Genomics.

[28] Wang F, Li L, Liu L, Li H, Zhang Y, Yao Y, Ni Z, Gao J (2012). High-throughput sequencing discovery of conserved and novel microRNAs in Chinese cabbage (*Brassica rapa* L. ssp. *pekinensis*). Mol Genet Genomics.

[29] Jia L, Zhang D, Qi X, Ma B, Xiang Z, He N (2014). Identification of the conserved and novel miRNAs in mulberry by high-throughput sequencing. PLoS ONE.

[30] Gao J, Yin F, Liu M, Luo M, Qin C, Yang A, Yang S, Zhang Z, Shen Y, Lin H, Pan G (2015). Identification and characterization of tobacco microRNA transcriptome using high-throughput sequencing. Plant Biol.

[31] Matousek J, Vrba L, Skopek J, Orctova L, Pesina K, Heyerick A, Baulcombe D, De Keukeleire D (2006). Sequence analysis of a “true” chalcone synthase (chs_H1) oligofamily from hop (*Humulus lupulus* L.) and PAP1 activation of chs_H1 in heterologous systems. J Agric Food Chem.

[32] Van Cleemput M, Cattoor K, De Bosscher K, Haegeman G, De Keukeleire D, Heyerick A (2009). Hop (*Humulus lupulus*)-derived bitter acids as multipotent bioactive compounds. J Nat Prod.

[33] Diener TO, Diener TO (1987). Biological properties. The Viroids.

[34] Daròs JA, Flores R (2004). *Arabidopsis thaliana* has the enzymatic machinery for replicating representative viroid species of the family Pospiviroidae. Proc Natl Acad Sci U S A.

[35] Sano T, Barba M, Li SF, Hadidi A (2010). Viroids and RNA silencing: mechanism, role in viroid pathogenicity and development of viroid-resistant plants. GM Crops.

[36] Wang Y, Shibuya M, Taneda A, Kurauchi T, Senda M, Owens RA, Sano T (2011). Accumulation of *Potato spindle tuber viroid*-specific small RNAs is accompanied by specific changes in gene expression in two tomato cultivars. Virology.

[37] Markarian N, Li HW, Ding SW, Semancik JS (2004). RNA silencing as related to viroid induced symptom expression. Arch Virol.

[38] Martinez G, Donaire L, Llave C, Pallás V, Gómez G (2010). High-throughput sequencing of Hop stunt viroid-derived small RNAs from cucumber leaves and phloem. Mol Plant Pathol.

[39] Jakse J, Radisek S, Pokorn T, Matousek J, Javornik B (2015). Deep-sequencing revealed *Citrus bark cracking viroid* (CBCVd) as a highly aggressive pathogen on hop. Plant Pathol.

[40] Hammann C, Steger G (2012). Viroid-specific small RNA in plant disease. RNA Biol.

[41] Diermann N, Matoušek J, Junge M, Riesner D, Steger G (2010). Characterization of plant miRNAs and small RNAs derived from *Potato spindle tuber viroid* (PSTVd) in infected tomato. Biol Chem.

[42] Blankenberg D, Gordon A, Kuster GV, Coraor N, Taylor J, Nekrutenko A, Galaxy Team (2010). Manipulation of FASTQ data with Galaxy. Bioinformatics.

[43] Lei J, Sun Y (2014). miR-PREFeR: an accurate, fast and easy-to-use plant miRNA prediction tool using small RNA-Seq data. Bioinformatics.

[44] Meyers BC, Axtell MJ, Bartel B, Bartel DP, Baulcombe D, Bowman JL, Cao X, Carrington JC, Chen X, Green PJ, Griffiths-Jones S, Jacobsen SE, Mallory AC, Martienssen RA, Poethig RS, Qi Y, Vaucheret H, Voinnet O, Watanabe Y, Weigel D, Zhu JK (2008). Criteria for annotation of plant microRNAs. Plant Cell.

[45] Natsume S, Takagi H, Shiraishi A, Murata J, Toyonaga H, Patzak J, Takagi M, Yaegashi H, Uemura A, Mitsuoka C, Yoshida K, Krofta K, Satake H, Terauchi R, Ono E (2015). The draft genome of hop (Humulus lupulus), an essence for brewing. Plant Cell Physiol.

[46] Mishra AK, Duraisamy GS, Matoušek J (2015). Discovering microRNAs and their targets in Plants. Crit Rev Plant Sci.

[47] Dai X, Zhao PX (2011). psRNATarget: a plant small RNA target analysis server. Nucleic Acids Res.

[48] Kantar M, Unver T, Budak H (2010). Regulation of barley miRNAs upon dehydration stress correlated with target gene expression. Funct Integr Genomics.

[49] Conesa A, Götz S, García-Gómez JM, Terol J, Talón M, Robles M (2005). Blast2GO: a universal tool for annotation, visualization and analysis in functional genomics research. Bioinformatics.

[50] Audic S, Claverie JM (1997). The significance of digital gene expression profiles. Genome Res.

[51] Kramer MF (2011). Stem-loop RT-qPCR for miRNAs. Curr Protoc Mol Biol.

[52] Bustin SA, Benes V, Garson JA, Hellemans J, Huggett J, Kubista M, Mueller R, Nolan T, Pfaffl MW, Shipley GL, Vandesompele J, Wittwer CT (2009). The MIQE guidelines: minimum information for publication of quantitative real-time PCR experiments. Clin Chem.

[53] Bologna NG, Mateos JL, Bresso EG, Palatnik JF (2009). A loop-to-base processing mechanism underlies the biogenesis of plant microRNAs miR319 and miR159. EMBO J.

[54] Rogers K, Chen X (2013). Biogenesis, turnover, and mode of action of plant microRNAs. Plant Cell.

[55] Petroni K, Kumimoto RW, Gnesutta N, Calvenzani V, Fornari M, Tonelli C, Holt BF, Mantovani R (2012). The promiscuous life of plant nuclear factor transcription factors. Plant Cell.

[56] Matoušek J, Kocábek T, Patzak J, Füssy Z, Procházková J, Heyerick A (2012). Combinatorial analysis of lupulin gland transcription factors from R2R3Myb, bHLH and WDR families indicates a complex regulation of chs_H1 genes essential for prenylflavonoid biosynthesis in hop (*Humulus lupulus* L.). BMC Plant Biol.

[57] Lu CG, Koroleva OA, Farrar JF, Gallagher J, Pollock CJ, Tomos AD (2002). Rubisco small subunit, chlorophyll a/b-binding protein and sucrose: fructan-6-fructosyl transferase gene expression and sugar status in single barley leaf cells in situ. Cell type specificity and induction by light. Plant Physiol.

[58] Dangl JL, Jones JD (2001). Plant pathogens and integrated defence responses to infection. Nature.

[59] Erickson FL, Dinesh-Kumar SP, Holzberg S, Ustach CV, Dutton M, Handley V, Corr C, Baker BJ (1999). Interactions between tobacco mosaic virus and the tobacco N gene. Philos Trans R Soc Lond B Biol Sci.

[60] Yu QB, Jiang Y, Chong K, Yang ZN (2009). AtECB2, a pentatricopeptide repeat protein, is required for chloroplast transcript accD RNA editing and early chloroplast biogenesis in *Arabidopsis thaliana*. Plant J.

[61] Moon YH, Chen L, Pan RL, Chang HS, Zhu T, Maffeo DM, Sung ZR (2003). EMF genes maintain vegetative development by repressing the flower program in Arabidopsis. Plant Cell.

[62] Schneider S (2015). Inositol transport proteins. FEBS Lett.

[63] Poteryaev D, Datta S, Ackema K, Zerial M, Spang A (2010). Identification of the switch in early-to-late endosome transition. Cell.

[64] Aldridge C, Maple J, Moller SG (2005). The molecular biology of plastid division in higher plants. J Exp Bot.

[65] Johnson CS, Kolevski B, Smyth DR (2002). TRANSPARENT TESTA GLABRA2, a trichome and seed coat development gene of Arabidopsis, encodes a WRKY transcription factor. Plant Cell.

[66] Minoia S, Carbonell A, Di Serio F, Gisel A, Carrington JC, Navarro B, Flores R (2014). Specific ARGONAUTES bind selectively small RNAs derived from *Potato spindle tuber viroid* and attenuate viroid accumulation in vivo. J Virol.

[67] Zavallo D, Debat HJ, Conti G, Manacorda CA, Rodriguez MC, Asurmendi S (2015). Differential mRNA accumulation upon early *Arabidopsis thaliana* infection with ORMV and TMV-Cg is associated with distinct endogenous small RNAs level. PLoS ONE.

[68] Brodersen P, Voinnet O (2006). The diversity of RNA silencing pathways in plants. Trends Genet.

[69] Zilberman D, Cao X, Johansen LK, Xie Z, Carrington JC, Jacobsen SE (2004). Role of Arabidopsis ARGONAUTE4 in RNA-directed DNA methylation triggered by inverted repeats. Curr Biol.

[70] Zhang BH, Pan XP, Cox SB, Cobb GP, Anderson TA (2006). Evidence that miRNAs are different from other RNAs. Cell Mol Life Sci.

[71] Axtell MJ, Snyder JA, Bartel DP (2007). Common functions for diverse small RNAs of land plants. Plant Cell.

[72] Li YF, Zheng Y, Addo-Quaye C, Zhang L, Saini A, Jagadeeswaran G, Axtell MJ, Zhang W, Sunkar R (2010). Transcriptome-wide identification of microRNA targets in rice. Plant J.

[73] Chen HM, Chen LT, Patel K, Li YH, Baulcombe DC, Wu SH (2010). 22-Nucleotide RNAs trigger secondary siRNA biogenesis in plants. Proc Natl Acad Sci U S A.

[74] Lu YD, Gan QH, Chi XY, Qin S (2008). Roles of microRNA in plant defense and virus offense interaction. Plant Cell Rep.

[75] Yu S, Galvão VC, Zhang YC, Horrer D, Zhang TQ, Hao YH, Feng YQ, Wang S, Schmid M, Wang JW (2012). Gibberellin regulates the Arabidopsis floral transition through miR156-targeted Squamosa promoter binding-like transcription factors. Plant Cell.

[76] Licausi F, Ohme-Takagi M, Perata P (2013). APETALA2/Ethylene responsive factor (AP2/ERF) transcription factors: mediators of stress responses and developmental programs. New Phytol.

[77] Curaba J, Singh MB, Bhalla PL (2014). miRNAs in the crosstalk between phytohormone signalling pathways. J Exp Bot.

[78] Hirsch S, Oldroyd GE (2009). GRAS-domain transcription factors that regulate plant development. Plant Signal Behav.

[79] Jiang SC, Mei C, Liang S, Yu YT, Lu K, Wu Z, Wang XF, Zhang DP (2015). Crucial roles of the pentatricopeptide repeat protein SOAR1 in Arabidopsis response to drought, salt and cold stresses. Plant Mol Biol.

[80] Liang W, Li C, Liu F, Jiang H, Li S, Sun J, Wu X, Li C (2009). The *Arabidopsis* homologs of CCR4-associated factor 1 show mRNA deadenylation activity and play a role in plant defence responses. Cell Res.

