# Supplementary material for: Identification and characterization of microRNAs in Humulus lupulus using high-throughput sequencing and their response to Citrus bark cracking viroid (CBCVd) infection
Source: BMC Genomics. 2016 Nov 15;17:919. doi: 10.1186/s12864-016-3271-4 (PMC5109749; doi:10.1186/s12864-016-3271-4)
Supplement: Additional file 1: Table S1. — Details of primers designed for RT-PCR amplification and 5′-RACE amplification of selected target genes. (DOC 49 kb) [file 12864_2016_3271_MOESM1_ESM.doc]

**Primers detail for qPCR analysis of 10 selected target genes**

| ***S.N*** | ***miRNA*** | ***Target Gene name*** | ***Forward primer (5′ to 3′)*** | ***Reverse primer (5′ to 3′)*** |
| --- | --- | --- | --- | --- |
| 1 | hop-miR156 | Squamosa promoter-binding-like protein 15 | ATCAAAGTGCTATTCCTACTGG | ATGACTAGCTGGGATACTCGT |
| 2 | hop-miR159c | Transcription factor GAMYB | AAATATTGCCACCCATTTCG | TATCACACGTAATTTCGGGA |
| 3 | hop-miR164a | NAC domain protein NAC1 | AGGCTACAACTCCAAGTCTC | AATTAGGTCATGGGTTTGGT |
| 4 | hop-miR167a-5p | Cysteine protease | TTCTTCCCACTAAAGATCTCC | GAGGAAGTTAGCACCTTCCA |
| 5 | hop-miR167c-5p | mediator of RNA pol II transcription subunit 26b | GGTCGCTAGATTACTGGAGG | TTCTACAACTGAACAACCTCTC |
| 6 | hop-miR171b | GRAS family transcription factor | GCAAAGCAGAAGATGTTGAG | CAAAGCCAATGAGTTGTGGA |
| 7 | hop-miR395a | pre-mRNA-splicing factor RNA helicase PRP1 | CTTCCGATTGTCCAATTCGA | CCAATGGCTCCAGATTTACTG |
| 8 | hop-miR827a | Glycerol-3-phosphate dehydrogenase | AGATTGATGAGATCCGTAGGC | TTTCAAATAAGCACAGCGTC |
| 9 | hop-miR02 | Disease resistance protein RPM1-like | GAGGTTGTCTAGCAGGAAGG | GCGTTCGAGATGATGTACAG |
| 10 | hop-miR41 | Cellulose synthase A catalytic subunit 4 | TTTACAGTGACATCAAAGGCAG | GGGTAGAGATGGAGAATGAC |
| 11 |  | Reference gene (Hl-GADPH) | ACCGGAGCCGACTTTGTTGTTG | TCGTACTCTGGCTTGTATTCCTTC |

**Primers detail for RLM-RACE analysis**

| ***S.N*** | ***Primer Name*** | ***Oligo sequence (5′ to 3′)*** |
| --- | --- | --- |
| 1 | hop-miR156 (inner) | TTGGGTGTTCATCAAGGAAT |
| 2 | hop-miR156 (outer) | AGTGATTGACAGTTCCACCA |
| 3 | hop-miR164a (inner) | ATTTGGTTCCATTTGGGTGCT |
| 4 | hop-miR164a (outer) | TCCATCAAGTTTGGTATCCCAT |
| 5 | hop-miR171b (inner) | GCAAAGCCAATGAGTTGTGGA |
| 6 | hop-miR171b (outer) | CTGAAGAACCGGTGAGATTTCA |
