# Supplementary material for: Identification and characterization of microRNAs in Humulus lupulus using high-throughput sequencing and their response to Citrus bark cracking viroid (CBCVd) infection
Source: BMC Genomics. 2016 Nov 15;17:919. doi: 10.1186/s12864-016-3271-4 (PMC5109749; doi:10.1186/s12864-016-3271-4)
Supplement: Additional file 2: Table S2. — Details of primers designed for RT-PCR amplification of 50 novel and 8 conserved miRNAs identified in this study. (DOC 143 kb) [file 12864_2016_3271_MOESM2_ESM.doc]

**Stem-loop primer detail for novel and conserved miRNAs in hop**.

| **S.No** | **Primer Name** | **Stem-loop RT primer (5′ to 3′)** |
| --- | --- | --- |
| 1 | SL-miR01 | GTCGTATCCAGTGCAGGGTCCGAGGTATTCGCACTGGATACGAC**TTCGGT** |
| 2 | SL-miR02 | GTCGTATCCAGTGCAGGGTCCGAGGTATTCGCACTGGATACGAC**TCTCTA** |
| 3 | SL-miR03 | GTCGTATCCAGTGCAGGGTCCGAGGTATTCGCACTGGATACGAC**AATCTG** |
| 4 | SL-miR04 | GTCGTATCCAGTGCAGGGTCCGAGGTATTCGCACTGGATACGAC**TGAATA** |
| 5 | SL-miR05 | GTCGTATCCAGTGCAGGGTCCGAGGTATTCGCACTGGATACGAC**ACAGTT** |
| 6 | SL-miR06 | GTCGTATCCAGTGCAGGGTCCGAGGTATTCGCACTGGATACGAC**AGAGTT** |
| 7 | SL-miR07 | GTCGTATCCAGTGCAGGGTCCGAGGTATTCGCACTGGATACGAC**GGCCGC** |
| 8 | SL-miR08 | GTCGTATCCAGTGCAGGGTCCGAGGTATTCGCACTGGATACGAC**AGTTTA** |
| 9 | SL-miR09 | GTCGTATCCAGTGCAGGGTCCGAGGTATTCGCACTGGATACGAC**TCAATA** |
| 10 | SL-miR10 | GTCGTATCCAGTGCAGGGTCCGAGGTATTCGCACTGGATACGAC**AGTCTA** |
| 11 | SL-miR11 | GTCGTATCCAGTGCAGGGTCCGAGGTATTCGCACTGGATACGAC**GCGAAT** |
| 12 | SL-miR12 | GTCGTATCCAGTGCAGGGTCCGAGGTATTCGCACTGGATACGAC**GTACCC** |
| 13 | SL-miR13 | GTCGTATCCAGTGCAGGGTCCGAGGTATTCGCACTGGATACGAC**TGTATA** |
| 14 | SL-miR14 | GTCGTATCCAGTGCAGGGTCCGAGGTATTCGCACTGGATACGAC**AATCTG** |
| 15 | SL-miR15 | GTCGTATCCAGTGCAGGGTCCGAGGTATTCGCACTGGATACGAC**CATATT** |
| 16 | SL-miR16 | GTCGTATCCAGTGCAGGGTCCGAGGTATTCGCACTGGATACGAC**TTTTTT** |
| 17 | SL-miR17 | GTCGTATCCAGTGCAGGGTCCGAGGTATTCGCACTGGATACGAC**GGTAGA** |
| 18 | SL-miR18 | GTCGTATCCAGTGCAGGGTCCGAGGTATTCGCACTGGATACGAC**GAGTTC** |
| 19 | SL-miR19 | GTCGTATCCAGTGCAGGGTCCGAGGTATTCGCACTGGATACGAC**TTCAGA** |
| 20 | SL-miR20 | GTCGTATCCAGTGCAGGGTCCGAGGTATTCGCACTGGATACGAC**GAGTTT** |
| 21 | SL-miR21 | GTCGTATCCAGTGCAGGGTCCGAGGTATTCGCACTGGATACGAC**TTTCGG** |
| 22 | SL-miR22 | GTCGTATCCAGTGCAGGGTCCGAGGTATTCGCACTGGATACGAC**CACCCG** |
| 23 | SL-miR23 | GTCGTATCCAGTGCAGGGTCCGAGGTATTCGCACTGGATACGAC**ACCACA** |
| 24 | SL-miR24 | GTCGTATCCAGTGCAGGGTCCGAGGTATTCGCACTGGATACGAC**CTCTCA** |
| 25 | SL-miR25 | GTCGTATCCAGTGCAGGGTCCGAGGTATTCGCACTGGATACGAC**AAAAAT** |
| 26 | SL-miR26 | GTCGTATCCAGTGCAGGGTCCGAGGTATTCGCACTGGATACGAC**TTCGGT** |
| 27 | SL-miR27 | GTCGTATCCAGTGCAGGGTCCGAGGTATTCGCACTGGATACGAC**ATGTTT** |
| 28 | SL-miR28 | GTCGTATCCAGTGCAGGGTCCGAGGTATTCGCACTGGATACGAC**CCTGTA** |
| 29 | SL-miR29 | GTCGTATCCAGTGCAGGGTCCGAGGTATTCGCACTGGATACGAC**CCTTTG** |
| 30 | SL-miR30 | GTCGTATCCAGTGCAGGGTCCGAGGTATTCGCACTGGATACGAC**AATAAT** |
| 31 | SL-miR31 | GTCGTATCCAGTGCAGGGTCCGAGGTATTCGCACTGGATACGAC**CAGAAT** |
| 32 | SL-miR32 | GTCGTATCCAGTGCAGGGTCCGAGGTATTCGCACTGGATACGAC**CAGTTT** |
| 33 | SL-miR33 | GTCGTATCCAGTGCAGGGTCCGAGGTATTCGCACTGGATACGAC**TCTAAA** |
| 34 | SL-miR34 | GTCGTATCCAGTGCAGGGTCCGAGGTATTCGCACTGGATACGAC**AGAGTT** |
| 35 | SL-miR35 | GTCGTATCCAGTGCAGGGTCCGAGGTATTCGCACTGGATACGAC**TCGGGT** |
| 36 | SL-miR36 | GTCGTATCCAGTGCAGGGTCCGAGGTATTCGCACTGGATACGAC**TTTGTT** |
| 37 | SL-miR37 | GTCGTATCCAGTGCAGGGTCCGAGGTATTCGCACTGGATACGAC**GTTTAC** |
| 38 | SL-miR38 | GTCGTATCCAGTGCAGGGTCCGAGGTATTCGCACTGGATACGAC**TGCAAA** |
| 39 | SL-miR39 | GTCGTATCCAGTGCAGGGTCCGAGGTATTCGCACTGGATACGAC**TCTCTA** |
| 40 | SL-miR40 | GTCGTATCCAGTGCAGGGTCCGAGGTATTCGCACTGGATACGAC**CGTTGA** |
| 41 | SL-miR41 | GTCGTATCCAGTGCAGGGTCCGAGGTATTCGCACTGGATACGAC**CTCTAA** |
| 42 | SL-miR42 | GTCGTATCCAGTGCAGGGTCCGAGGTATTCGCACTGGATACGAC**TCTCTA** |
| 43 | SL-miR43 | GTCGTATCCAGTGCAGGGTCCGAGGTATTCGCACTGGATACGAC**GGTGAT** |
| 44 | SL-miR44 | GTCGTATCCAGTGCAGGGTCCGAGGTATTCGCACTGGATACGAC**GTCAAT** |
| 45 | SL-miR45 | GTCGTATCCAGTGCAGGGTCCGAGGTATTCGCACTGGATACGAC**AGTTCA** |
| 46 | SL-miR46 | GTCGTATCCAGTGCAGGGTCCGAGGTATTCGCACTGGATACGAC**ACGGTG** |
| 47 | SL-miR47 | GTCGTATCCAGTGCAGGGTCCGAGGTATTCGCACTGGATACGAC**ATCAAA** |
| 48 | SLmiR48 | GTCGTATCCAGTGCAGGGTCCGAGGTATTCGCACTGGATACGAC**GTTGAA** |
| 49 | SL-miR49 | GTCGTATCCAGTGCAGGGTCCGAGGTATTCGCACTGGATACGAC**AGGTTC** |
| 50 | SL-miR156 | GTCGTATCCAGTGCAGGGTCCGAGGTATTCGCACTGGATACGAC**GTGCTC** |
| 51 | SL-miR159c | GTCGTATCCAGTGCAGGGTCCGAGGTATTCGCACTGGATACGAC**GGAGCT** |
| 52 | SL-miR164a | GTCGTATCCAGTGCAGGGTCCGAGGTATTCGCACTGGATACGAC**TGCACG** |
| 53 | SL-miR167a-5p | GTCGTATCCAGTGCAGGGTCCGAGGTATTCGCACTGGATACGAC**TAGATC** |
| 54 | SL-miR167c-5p | GTCGTATCCAGTGCAGGGTCCGAGGTATTCGCACTGGATACGAC**GCAGAT** |
| 55 | SL-miR171b | GTCGTATCCAGTGCAGGGTCCGAGGTATTCGCACTGGATACGAC**GATATT** |
| 56 | SL-miR395a | GTCGTATCCAGTGCAGGGTCCGAGGTATTCGCACTGGATACGAC**GGAGCT** |
| 57 | SL-miR827a | GTCGTATCCAGTGCAGGGTCCGAGGTATTCGCACTGGATACGAC**TGTTTG** |
| 58 | SL- U6 | GTGCAGGGTCCGAGGTTTTGGACCATTTCTCGAT |

**Forward primer detail for novel and conserved miRNAs** in hop

| **S.No** | **Primer name** | **Forward primer (5′ to 3′)** |
| --- | --- | --- |
| 1 | F-miR01 | GCTGCCCGCCTCTGAATAAATT |
| 2 | F-miR02 | TGCCAGCTTTCTGTTCGAC |
| 3 | F-miR03 | TGCCAGCCTTGGTAC CCTAA |
| 4 | F-miR04 | GACCGTGGCTTTTTGGCACTG |
| 5 | F-miR05 | CAGCGTAGCCCGAAACATGGT |
| 6 | F-miR06 | CGCCCGCTAGTAATTGACTGT |
| 7 | F-miR07 | CGCCCGCTACTAATGTCTCTG |
| 8 | F-miR08 | TGCCAGCCCTAGTTTTCGGT |
| 9 | F-miR09 | TGACCAGCCGGGTACCATATG |
| 10 | F-miR10 | GCCGCCCCCGTATAAAATAGTT |
| 11 | F-miR11 | TATTGCGGCTCGGTGAACTCT |
| 12 | F-miR12 | CGCCCGCCGCAATTAAATTTGA |
| 13 | F-miR13 | TGCGAGCCCTGCTCTAAATAAC |
| 14 | F-miR14 | TGCCAGCCTTTGGTACCCTAA |
| 15 | F-miR15 | TGCCACCGTTTAGATCTTCC |
| 16 | F-miR16 | TGAGGAGCCGAAACGTGTAGAT |
| 17 | F-miR17 | GTCTCCGCCCTCTCGAAATAC |
| 18 | F-miR18 | CCGTGCACCGGAAAACAGATTA |
| 19 | F-miR19 | CCGTGCACCGTAAACTATTCGA |
| 20 | F-miR20 | CCCTGCAGCGACATATTTGGTA |
| 21 | F-miR21 | CCCTGCCCCGTGAATAGTTTAC |
| 22 | F-miR22 | CGGTGGGGCGAGGATAAATCTA |
| 23 | F-miR23 | AGGCGGGGCGGTTTAAATTTTA |
| 24 | F-miR24 | GCATCATTCACGGGTCGAGA |
| 25 | F-miR25 | GGGCGATTTTGGATTCAGGG |
| 26 | F-miR26 | CGCCCGCTATCCGAATAGTTT |
| 27 | F-miR27 | GACCTTGACTTCGACGCTGAAC |
| 28 | F-miR28 | CCTGGCGTGATTGGTCAAGT |
| 29 | F-miR29 | GTTCCGCCTATCTGTAGCCAG |
| 30 | F-miR30 | GCTCCGCTTTTTGGCACTG |
| 31 | F-miR31 | CCCGCCGTGCAAATTTTCAG |
| 32 | F-miR32 | TATCGCCGAGCCGAAAACAA |
| 33 | F-miR33 | CACCCGCCGTGTGTATATTGT |
| 34 | F-miR34 | CGCTGCCGTGTAATTGACTGT |
| 35 | F-miR35 | CCGCTGCCGCTTAAAATAGAAC |
| 36 | F-miR36 | CCGCCGCCTCTGAATTATCA |
| 37 | F-miR37 | ATCGTCGCCCTAGTTTTCGGT |
| 38 | F-miR38 | ATCGTCGCCACATGAGCAAT |
| 39 | F-miR39 | ATCCCTGCCTTTCTGTTCGAC |
| 40 | F-miR40 | ATCCCTGCCTCAGAAACTCCA |
| 41 | F-miR41 | GCCGTCCGCTATGTACATTGTA |
| 42 | F-miR42 | GCGTCCGTCTTTCTGATCGAC |
| 43 | F-miR43 | GACTGATATTCGGGGCTTTGGG |
| 44 | F-miR44 | GCCGCCTGATTGACAGTTTGAT |
| 45 | F-miR45 | TGGAGGATCTAGTTTAGGGGCC |
| 46 | F-miR46 | CAGCGAGATCAAACCGTCCA |
| 47 | F-miR47 | CAGCAGCGACCCTAGACTTAG |
| 48 | FmiR48 | CAGCAGCGGGAGACTTTGT |
| 49 | F-miR49 | CGAGACAGCCGTGGAAAACAT |
| 50 | F-miR156 | CGCAGACCGTGACAGAAGAGA |
| 51 | F-miR159c | GCCTGTCCGCTTGGATTGAAG |
| 52 | F-miR164a | GAATGTCCGTGGAGAAGCAGG |
| 53 | F-miR167a-5p | GAATGTCCGTGAAGCTGCCAG |
| 54 | F-miR167c-5p | GAATGTCCGTGAAGCTGCCAG |
| 55 | F-miR171b | CAGTGTCCGTGATTGAGCCGT |
| 56 | F-miR395a | GCCTGTCCGTGAAGTGTTTGG |
| 57 | F-miR827a | CCAGTGCAGGGTCCGAGGTA |
| 58 | F-U6 | GGAACGATACAGAGAAGATTAGCA |

Universal reverse primer

| **S.No** | **Primer name** | **Reverse primer (5′ to 3′)** |
| --- | --- | --- |
| 1 | miRNA-URP | CCAGTGCAGGGTCCGAGGTA |
| 2 | U6-URP | GTGCAGGGTCCGAGGT |
