# Supplementary figures and images for: Identification and characterization of microRNAs in Humulus lupulus using high-throughput sequencing and their response to Citrus bark cracking viroid (CBCVd) infection
Source: BMC Genomics. 2016 Nov 15;17:919. doi: 10.1186/s12864-016-3271-4 (PMC5109749; doi:10.1186/s12864-016-3271-4)

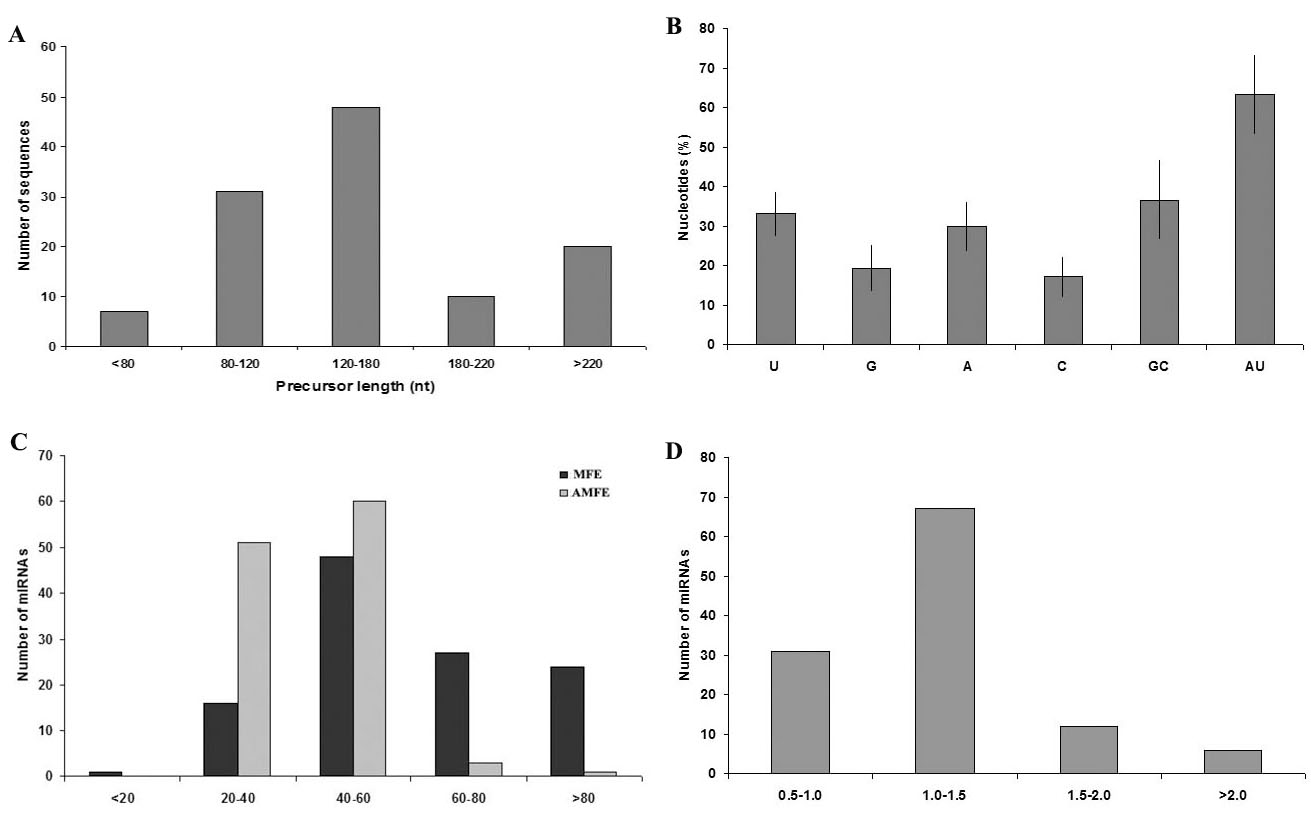

Supplement: Additional file 4: Figure S1. — Characterization of miRNAs identified in hop. (A) length distribution of pre-miRNAs; (B) distribution of nucleotides; (C) MFE and AMFE; and MFEI (D). (JPG 64 kb) [file 12864_2016_3271_MOESM4_ESM.jpg]

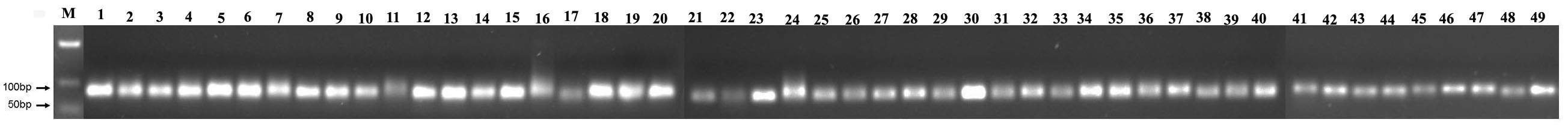

Supplement: Additional file 5: Figure S2. — End-point PCR validation by agarose gel electrophoresis for amplicons of the 50 novel miRNAs identified in this study. Small RNA was isolated from CBCVd-infected leaf and amplified with miRNA specific forward primer and a universal reverse primer. (JPG 60 kb) [file 12864_2016_3271_MOESM5_ESM.jpg]

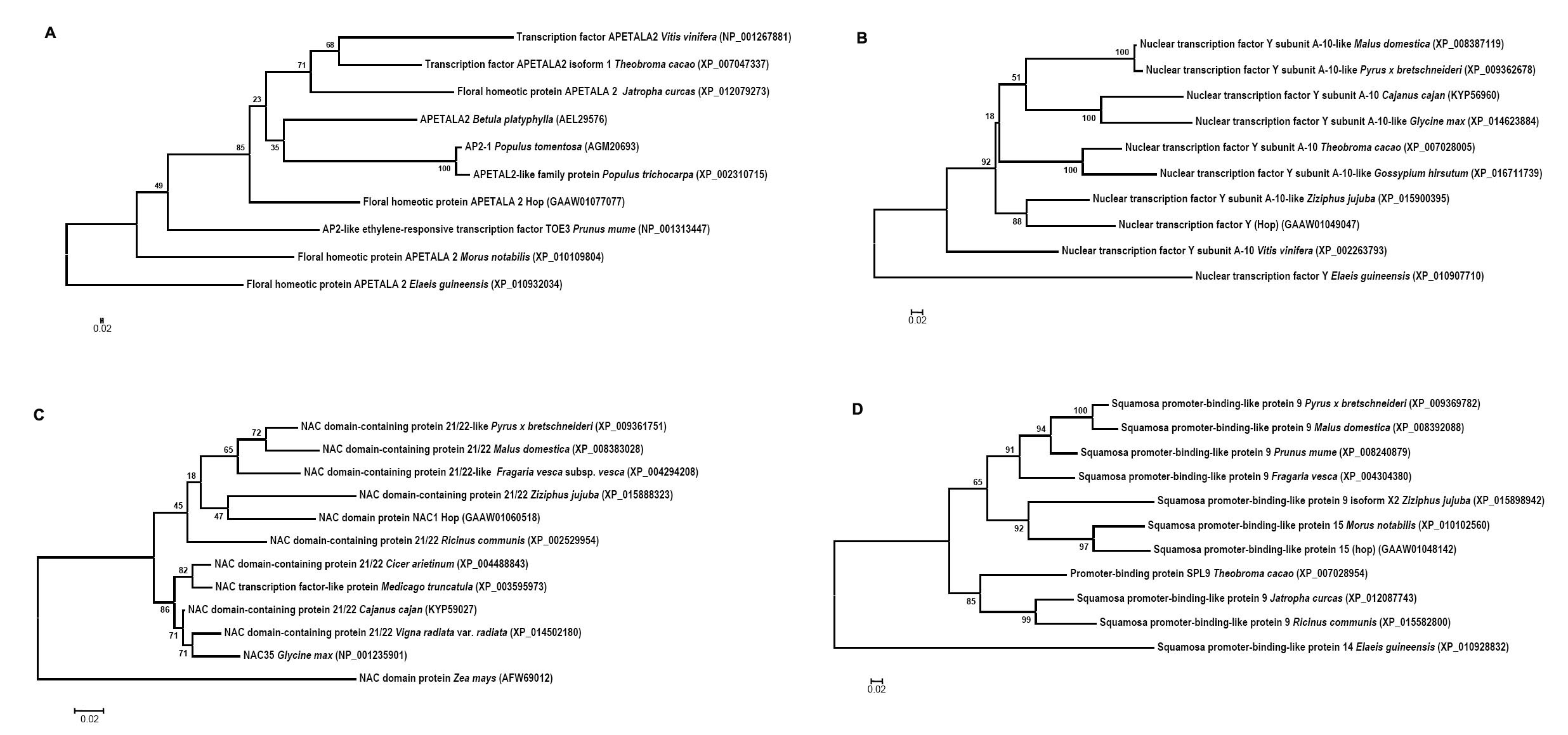

Supplement: Additional file 8: Figure S3. — Phylogenetic tree of APETALA 2 (A), NF-Y (B), NAC (C), SPL (D) transcriptional factor in hop. Multiple sequence alignment of the full length of protein sequences was performed using the ClustalW program. Phylogenetic trees were constructed via the maximum composite likelihood substitution model using MEGA (version 6.0). Bootstrapping was performed 1000 times to obtain support values for each branch. The scale bar represents the amino acid substitution rate. The tree has been rooted using the single representative of monocotyledonous plant. (JPG 324 kb) [file 12864_2016_3271_MOESM8_ESM.jpg]

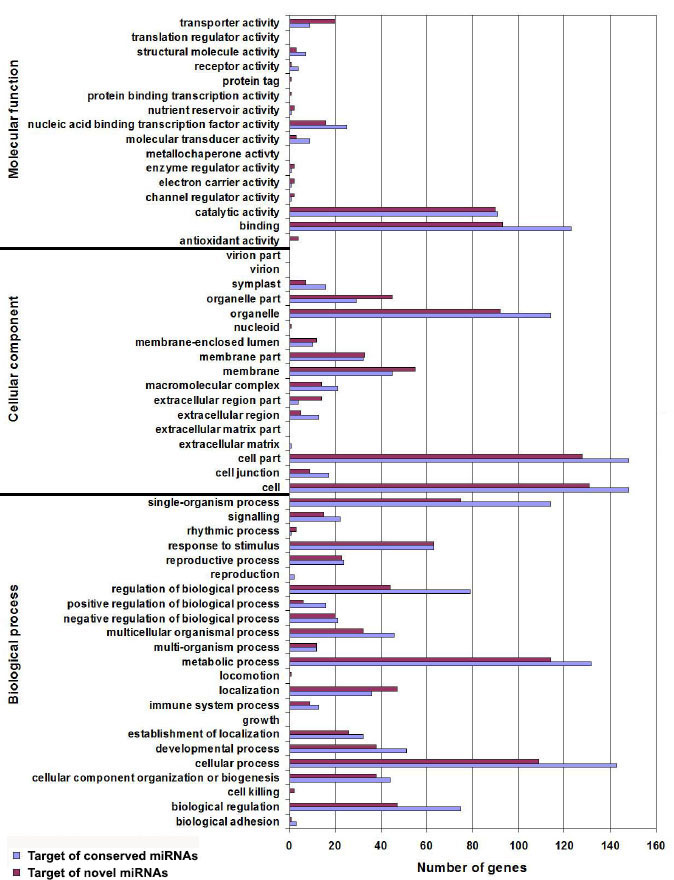

Supplement: Additional file 9: Figure S4. — Distribution of GO categories for the predicted target genes of conserved and novel miRNAs identified in hop. The vertical axis represents the GO category, including biological processes, cellular components and molecular functions and the horizontal axis represents the number of genes. (JPG 158 kb) [file 12864_2016_3271_MOESM9_ESM.jpg]
